# Role of Endoscopic Ultrasonography in Management of Pancreaticobiliary Cancers: Recent Trends and Advances

**DOI:** 10.3390/cancers18121864

**Published:** 2026-06-07

**Authors:** Shivangini Duggal, Mutaz Kalas, Mohamed H. Eldesouki, M. Ammar Kalas, Sherif E. Elhanafi

**Affiliations:** 1 Department of Medicine, Texas Tech University Health Sciences Center, El Paso, TX 79905, USA; mukalas@ttuhsc.edu; 2 Department of Internal Medicine, New York Medical College at Saint Michael’s Medical Center, Newark, NJ 07102, USA; 3 Division of Gastroenterology, Texas Tech University Health Sciences Center, El Paso, TX 79905, USA; mkalas@ttuhsc.edu (M.A.K.);; 4 Division of Gastroenterology, University of Texas at Tyler School of Medicine, Tyler, TX 75708, USA

**Keywords:** endoscopic ultrasound, pancreatic cancer, gall bladder cancer, cholangiocarcinoma

## Abstract

In this review, we explore the evolving role of endoscopic ultrasound (EUS) in diagnosing and managing pancretobiliary malignancies. For solid pancreatic lesions, techniques like fine-needle biopsy (FNB), contrast-enhanced EUS (CE-EUS), and macroscopic on-site evaluation (MOSE) improve sample quality and diagnostic accuracy. In cystic pancreatic lesions, fine-needle aspiration (FNA), molecular testing, and confocal laser endomicroscopy (nCLE) aid in distinguishing benign from malignant cysts. For cholangiocarcinoma, EUS guided sampling is more accurate than CT in assessing distal lesions and lymph node metastases, while combining EUS with magnetic resonance cholangiography (MRC) enhances diagnostic sensitivity. In gallbladder cancer, EUS surpasses CT and MRI in detecting lymphadenopathy and staging tumors. EUS-FNB (Fine needle biopsy) improves biopsy accuracy, especially for unresectable cases. These advancements highlight EUS as a critical tool for early cancer detection, staging, and tissue acquisition. Beyond diagnosis, EUS plays a pivotal therapeutic role in managing complications such as malignant biliary obstruction and gastric outlet obstruction, offering minimally invasive alternatives like EUS-guided biliary drainage and gastroenterostomy with high clinical success and improved patient outcomes.

## Simple Summary

This review highlights recent trends and advances in endoscopic ultrasound (EUS) for pancreatobiliary malignancies. In solid and cystic pancreatic lesions, contemporary FNB needles, optimized suction techniques, MOSE, contrast-enhanced EUS, detective flow imaging, elastography, and artificial intelligence enhance diagnostic accuracy, while EUS-guided tissue acquisition supports precision-medicine applications including comprehensive genomic profiling. The review also addresses EUS-based screening, celiac plexus interventions for pain, and tumor ablation—notably EUS-RFA for pancreatic neuroendocrine tumors. In biliary and gallbladder cancer, EUS complements ERCP-based sampling in line with recent ESGE guidelines. Therapeutic EUS, through biliary drainage and gastroenterostomy, provides minimally invasive alternatives for malignant obstruction.

## 1. Introduction

Endoscopic ultrasonography (EUS) is a widely utilized diagnostic and therapeutic modality in the evaluation and management of pancreatobiliary diseases. Introduced into clinical practice in the 1980s, EUS has revolutionized the approach to pancreatic and biliary disorders by providing high-resolution, real-time imaging of the gastrointestinal (GI) tract and surrounding structures [1]. Initially developed as a diagnostic tool, EUS has undergone significant advancements, evolving into an essential technique for tissue acquisition and therapeutic interventions [2].

Early applications of EUS focused primarily on diagnostic imaging, particularly in detecting small pancreatic neoplasms where it demonstrated superior sensitivity compared to other imaging modalities such as computed tomography (CT), magnetic resonance imaging (MRI), and transabdominal ultrasound [3]. The advent of contrast-enhanced EUS (CE-EUS) and EUS elastography has enhanced diagnostic accuracy, while interventional EUS has emerged as a minimally invasive alternative to conventional surgical procedures [4].

Therapeutic EUS applications have continued to grow, demonstrating high technical and clinical success rates with a low incidence of adverse events. Advanced EUS-guided procedures, such as gastroenterostomy creation, gallbladder drainage, angiotherapy, portal vein sampling, and EUS-guided endoscopic retrograde cholangiopancreatography (ERCP) in patients with surgically altered anatomy, have solidified its role in multidisciplinary management. The integration of EUS into oncologic and surgical decision-making has proven instrumental in optimizing patient outcomes while providing a minimally invasive approach [5].

EUS examinations are performed using two primary types of echoendoscopes: radial and linear. The radial echoendoscope generates a 360-degree image perpendicular to the scope’s axis, similar to CT imaging, making it ideal for diagnostic staging, particularly in assessing esophageal, gastric, and rectal malignancies as well as subepithelial lesions. This type of probe is especially useful for evaluating the gut wall structure and adjacent lymph nodes, making it the preferred choice for initial diagnostic evaluations [6,7]. In contrast, the linear echoendoscope produces images parallel to the scope’s axis, facilitating real-time tracking of needle punctures during fine-needle aspiration (FNA) or fine-needle biopsy (FNB). Linear echoendoscopes are crucial for therapeutic procedures, including drainage of pancreatic fluid collections, celiac plexus neurolysis, targeted biopsies, EUS-guided biliary drainage, and EUS-guided vascular access. They are equipped with an instrument channel, ranging from 2.8 to 3.8 mm in diameter. Additionally, the elevator mechanism in linear echoendoscopes enables greater control and precision in needle manipulation [8,9]. Miniature probes (mini-probes) are also available and can be inserted through the working channel of a standard endoscope. These probes provide high-frequency imaging and are particularly useful for evaluating intraductal structures, such as the pancreatic and biliary ducts during ERCP [10].

**Figure 1:**
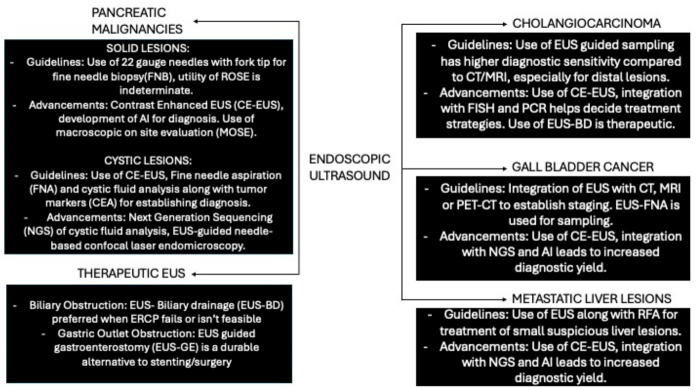
The image provides an overview of the evolving role of endoscopic ultrasound (EUS) in pancreatobiliary malignancies. EUS serves as a comprehensive platform for diagnosis, staging, tissue acquisition, and therapy. In pancreatic lesions, EUS-guided fine-needle biopsy (FNB), contrast-enhanced EUS (CE-EUS), artificial intelligence (AI), and advanced cystic fluid analysis improve diagnostic accuracy. In cholangiocarcinoma and gallbladder cancer, EUS enhances detection, nodal staging, and tissue acquisition, with CE-EUS, FISH/PCR, and next-generation sequencing (NGS) further refining diagnostic yield. EUS also plays a key role in detecting and sampling metastatic liver lesions, particularly small lesions missed by cross-sectional imaging. Beyond diagnosis, therapeutic EUS has emerged as a minimally invasive alternative for managing complications such as malignant biliary obstruction via EUS-guided biliary drainage (EUS-BD) and gastric outlet obstruction via EUS-guided gastroenterostomy (EUS-GE).

With continuous advancements in technology and technique, EUS remains at the forefront of pancreatobiliary cancer diagnosis and intervention. This review will explore the evolving role of diagnostic and interventional EUS in the management of pancreaticobiliary malignancies (Figure 1).

EUS FOR PANCREATIC CANCER

Pancreatic cancer, is a highly fatal malignancy with a lifetime incidence of approximately 1.6% and a 5-year survival rate of 10% [11]. In 2020, it accounted for 3% of all newly diagnosed cancers and 8% of cancer-related deaths in the United States [12]. Early-stage detection is linked to significantly better outcomes, with stage 0 pancreatic cancer (carcinoma in situ) having a 10-year survival rate of 93%, while stage I disease is associated with a 5-year survival rate of 34% to 39% [13]. However, nearly 80% of patients present with advanced, unresectable disease [14].

The proportion of solid versus cystic lesions in pancreatic cancer varies, with solid tumors being more prevalent. Pancreatic ductal adenocarcinoma (PDAC) is the most common malignancy, comprising approximately 95% of all pancreatic cancers. Neuroendocrine tumors also typically appear as solid masses [15]. A study by Kosmal et al. reported that 8% of PDACs exhibited a cystic morphology, while another study found that 10% of pancreatic neuroendocrine tumors were cystic [16,17]. The American Society of Gastrointestinal Endoscopy (ASGE) provides distinct guidelines for the diagnosis and staging of solid versus cystic pancreatic lesions, reflecting their differing clinical implications.

### 1.1. Diagnosis and Staging of Solid Pancreatic Lesions

EUS demonstrates high sensitivity and specificity in evaluating solid pancreatic lesions (SPLs), including those smaller than 20 mm [18,19]. However, its ability to reliably distinguish between benign and malignant lesions remains limited. To address this limitation, advancements such as CE-EUS and EUS elastography have been developed to enhance the diagnostic accuracy of EUS. The diagnostic process typically begins with B-mode EUS, which serves as the initial imaging modality to locate and characterize lesions. B-mode allows for the identification of lesions based on echogenicity—classifying them as hypoechoic, hyperechoic, or heterogeneous—but does not provide definitive information regarding malignancy [20]. Once a lesion is identified, FNB is performed for tissue sampling. CE-EUS and EUS elastography are then employed as adjunct techniques to further assess the malignancy potential of the lesion [21]. Among these modalities, CE-EUS has demonstrated superior performance in detecting pancreatic cancer compared to conventional B-mode EUS [20].

CE-EUS is classified into two subtypes: contrast-enhanced Doppler EUS and contrast-enhanced harmonic EUS (CEH-EUS). Contrast-enhanced Doppler EUS utilizes Doppler ultrasound technology combined with an intravenous contrast agent to assess vascular flow within pancreatic lesions. This approach provides real-time blood flow analysis, improving the detection of neoplastic vascular patterns [22]. However, its diagnostic accuracy may be limited by interference from background signals and motion artifacts. In contrast, CEH-EUS employs air-filled microbubble contrast agents that generate higher intensity signals within blood vessels compared to surrounding tissues. This technique enhances sensitivity and specificity in identifying malignancies by allowing selective visualization of vascular structures. CEH-EUS has been shown to improve overall diagnostic accuracy compared to conventional EUS, particularly in cases where standard imaging techniques yield inconclusive results [23].

Several studies have evaluated the diagnostic performance of CE-EUS compared to conventional EUS [24]. A recent meta-analysis reported that hypo-enhancement observed on CEH-EUS demonstrated a pooled sensitivity and specificity of 94% and 89%, respectively, for detecting malignancies [22]. The ability of CEH-EUS to provide clearer lesion delineation has proven beneficial in cases where conventional EUS is insufficient for distinguishing benign from malignant tumors [25]. In a prospective multicentric study, Omoto et al. examined the diagnostic utility of CE-EUS in PDAC, specifically assessing irregular periphery patterns and late-phase hypo-enhancement. Their findings demonstrated that CE-EUS significantly improved reproducibility in diagnosing PDAC, yielding a diagnostic accuracy of 85.8% compared to 78.9% with tissue-harmonic EUS (*p* < 0.001) [26]. Furthermore, Iwasa et al. investigated the combined use of qualitative analysis through CEH-EUS, which evaluates tumor microvascular patterns, and quantitative analysis using time-intensity curve measurements. Their study revealed that malignant lesions exhibited significantly shorter time-to-peak values compared to benign lesions (*p* = 0.0009). Integrating both approaches with EUS-guided sampling further enhanced diagnostic accuracy in evaluating SPLs [27].

Beyond its role in diagnosis, CEH-EUS has shown potential in predicting prognosis and assessing tumor aggressiveness. Ishikawa et al. reported that hypo-enhancement on CEH-EUS served as an indicator of aggressive pancreatic neuroendocrine tumors (pNETs), with reported sensitivity, specificity, positive predictive value, negative predictive value, and overall accuracy of 94.7%, 100%, 100%, 96.6%, and 97.9%, respectively [28]. These findings were further supported by Constantin et al., who conducted a pilot study on 30 patients to evaluate the prognostic implications of CEH-EUS in PDAC and pNETs. The authors noted that lower peak enhancement and reduced wash-in area under the curve on CEH-EUS were significantly associated with poorer survival outcomes in PDAC patients [29]. CE-EUS also plays a significant role in the staging of pancreatic cancers by providing enhanced visualization of tumor boundaries and vascular involvement. This technique is particularly useful in assessing the extent of local invasion and lymph node metastases, facilitating more precise staging and treatment planning [30].

EUS elastography is an advanced imaging technique that assesses tissue stiffness by measuring strain changes in response to compression. This method uses conventional B-mode imaging combined with specialized software to detect and display variations in tissue elasticity in real time [31]. Multiple meta-analyses have evaluated the diagnostic utility of EUS elastography for differentiating malignant pancreatic tumors. Reported sensitivity for detecting malignancy ranges from 92% to 98%. A meta-analysis by Zhang et al. reported a diagnostic sensitivity of 87% for PDAC. However, specificity remains lower, varying between 67% and 76%, primarily due to a high number of false-positive results [32,33,34]. In comparison, CE-EUS has been shown to offer superior specificity, making elastography a complementary rather than standalone tool in pancreatic lesion assessment. Despite promise, EUS elastography is highly operator-dependent with variable image selection/interpretation. Compression-related deformation and adjacent-tissue effects can distort stiffness and misclassify lesions. Recent studies show no clear improvement in sample adequacy or diagnostic yield over conventional EUS, so its incremental value remains uncertain [35,36,37].

Beyond CE-EUS and elastography, a new generation of non-contrast microvascular imaging modalities has recently emerged, addressing the main limitations of conventional color/power Doppler EUS, namely, poor sensitivity for slow-flow signals and degradation by motion artifact [38]. Detective flow imaging (DFI), together with superb microvascular imaging (SMI) and B-flow imaging, relies on an algorithm that separates low-velocity blood-flow signals from overlapping tissue motion artifact rather than suppressing them with a conventional wall filter, thereby preserving the visualization of minute and slow-flow vessels at a markedly higher frame rate and spatial resolution [39]. In a head-to-head comparison of 107 patients with solid pancreatic lesions, Mulqui et al. reported that DFI-EUS correlated strongly with CE-EUS for the assessment of intralesional microvascularity, with a sensitivity of 88.5%, specificity of 98.2%, positive predictive value of 97.9%, and negative predictive value of 90% [40]. Yamashita et al., in a prospective comparison of DFI-EUS, directional power Doppler (eFLOW)-EUS, and CEH-EUS in 90 patients, showed that DFI-EUS achieved a sensitivity, specificity, and accuracy for pancreatic cancer of 93%, 82%, and 88%, respectively, significantly superior to eFLOW-EUS (*p* = 0.005) and comparable to CH-EUS [41]. Similarly, Miwa et al., in 104 patients with pathologically confirmed solid pancreatic lesions, demonstrated that DFI visualized intralesional vessels in 96% of cases versus only 27% with eFLOW, with a significantly higher frame rate (median 43 vs. 12 fps; *p* < 0.01) and a diagnostic accuracy of 88% for pancreatic cancer [42]. Because DFI does not require intravenous contrast administration, it carries no risk of contrast-related adverse events, can be repeated or extended without a limited imaging window, and allows simultaneous evaluation of multiple lesions in the same session. Current limitations include platform dependence (DFI is presently integrated only into the latest-generation ARIETTA systems), reduced resolution at greater distances from the transducer, and still-limited evidence base derived predominantly from single-center retrospective studies [43]. Nonetheless, the available data support DFI and related microvascular imaging techniques as promising, contrast-free adjuncts to CE-EUS for the characterization of pancreatic lesions, warranting prospective multicenter validation.

Artificial intelligence (AI) is increasingly being integrated into EUS, AI-driven deep-learning algorithms have been trained to analyze EUS images, improving the detection and characterization of pancreatic lesions [44,45]. Most AI for EUS is supervised deep learning trained on labeled images to detect/characterize pancreatic lesions. A recent study on EUS-nCLE used a two-step pipeline (structure detection → dysplasia grading) with a CNN/ViT model that was self-supervised pre-trained (DINO-v2) and then supervised fine-tuned on labeled frames. The AI alone reached AUC ~0.70; combined with revised Fukuoka criteria it achieved AUC ~0.85 with ~78% sensitivity and specificity, outperforming experts and reducing interobserver variability [46]. A systematic review reported that AI-assisted EUS achieved a pooled sensitivity and specificity of 91% for detecting pancreatic malignancies [47]. AI models also show promise in distinguishing chronic pancreatitis from pancreatic cancer and optimizing FNA by identifying the most suitable biopsy sites. Emerging AI systems, such as BP MASTER^®^ and deep-learning convolutional neural networks, assist in real-time station recognition, benefiting trainees and improving procedural consistency [48,49].

A meta-analysis by Shin et al. demonstrated that when EUS elastography was combined with CE-EUS, there was a pooled sensitivity of 84% and specificity of 85%, however these techniques are not approved by FDA for diagnosis of pancreatic malignancies and histological diagnosis with EUS guided tissue acquisition remains the gold standard [20].

### 1.2. Diagnosis and Staging of Cystic Pancreatic Lesions

Pancreatic cystic lesions (PCLs) are increasingly detected (13.5–49.1%) due to the widespread use of cross-sectional imaging [50]. PCLs include: serous cystadenomas (SCNs), intraductal papillary mucinous neoplasms (IPMNs), mucinous cystic neoplasms (MCNs), pancreatic neuroendocrine tumors (pNETs), solid pseudopapillary tumors (SPTs), and pancreatic pseudocysts [51]. Differentiating features on EUS include cyst size, wall thickness, presence of septations, mural nodules, calcifications, and communication with the main pancreatic duct (MPD) or its branches [52]. While most PCLs are benign, a subset carries malignant potential. For instance, main duct intraductal papillary mucinous neoplasms (MD-IPMNs) demonstrate a malignancy risk ranging from 38 to 68%, branch duct intraductal papillary mucinous neoplasms (BD-IPMNs) have an intermediate malignancy potential of 15–17%, and MCNs have a malignancy risk of approximately 10% [53,54]. pNETs and SPTs exhibit cystic components in 8.02% and 33.33% of cases, respectively, with malignant potential varying from 6 to 31% for pNETs and around 10% for SPTs [55]. Pancreatic pseudocysts, commonly seen in pancreatitis, have no inherent malignant risk but can mimic neoplastic cysts on imaging [56].

EUS features concerning for malignancy include large cyst size (>3 cm), thick septations, thick cyst walls, bile duct obstruction, dilated MPD, and the presence of mural nodules [57]. A meta-analysis of 41 studies found that mural nodules (OR: 9.3), MPD dilation (OR: 7.27), and main duct involvement in IPMNs (OR: 4.7) are independent risk factors for malignant transformation [58]. Another meta-analysis reported that mural nodule size is a significant predictor of malignancy, with studies identifying cutoff values of 5 mm, 7 mm, and 10 mm for predicting high-grade dysplasia or invasive carcinoma [59]. Additionally, Iwaya et al. found that a septal thickness cutoff of 2.5 mm on EUS accurately predicted malignant transformation in IPMNs [60]. While early reports showed an accuracy of 92–96% in differentiating cystic lesions, later studies by Brugge et al. highlighted the limitations of EUS morphology alone, reporting sensitivities, specificities, and accuracies of 56.1%, 45.4%, and 50.9%, respectively [61]. CE-EUS has significantly improved the ability to assess PCLs, particularly for identifying mural nodules [62]. European Society of Gastrointestinal Endoscopy (ESGE) recommends CE-EUS for evaluating mural nodules, as hyperenhancement is strongly associated with malignancy [63]. A meta-analysis of eight studies including 320 PCLs found that CE-EUS had a pooled sensitivity and specificity of 97.0% and 90.4%, respectively, for diagnosing mural nodules with high-grade dysplasia or invasive carcinoma [22] (Table 1). Moreover, CE-EUS-guided FNA targeting enhanced mural nodules demonstrated a high rate of dysplasia or malignancy detection.

Cystic fluid analysis has become an essential tool in distinguishing benign from malignant PCLs. Cyst fluid analysis using intracystic glucose (≤25 mg/dL) shows superior sensitivity (88.1%) and specificity (91.2%) over CEA [64]. KRAS and GNAS mutations further enhance diagnostic accuracy (97%), sensitivity (94%), and specificity (91%) for IPMNs and MCNs diagnosis [65]. Next gene sequencing (NGS) detects advanced malignancies with 89% sensitivity and 98% specificity. SCNs with TP53/TERT mutations and pNETs with ≥3 gene loss show aggressive behavior. It has also been noted that SCNs with TP53/TERT mutations exhibited interval growth, while pNETs with loss of heterozygosity of ≥3 genes tended to have distant metastasis [66]. EUS-guided needle-based confocal laser endomicroscopy (nCLE) has emerged as an advanced technique for in vivo microscopic evaluation of cyst wall epithelium. This approach provides real-time, high-resolution imaging, allowing precise differentiation of mucinous from non-mucinous cysts. Initial retrospective studies demonstrated that nCLE significantly improved the detection of serous cystadenomas compared to conventional cyst fluid analysis [67]. nCLE demonstrates high accuracy in classifying PCLs, with sensitivity, specificity, and accuracy rates of 98%, 94%, and 97%, respectively. Compared to endoscopic ultrasound-guided through the needle biopsy (EUS-TTNB), it offers a higher diagnostic yield (85% vs. 74%, *p* < 0.001) with similar safety. Additionally, papillary epithelial width and darkness observed via EUS-guided nCLE accurately predict high-grade dysplasia in IPMNs [68,69]. The role of AI is also expanding for the diagnosis of PCLs. A recent pilot study utilizing 5505 EUS images from 28 PCLs demonstrated the ability to accurately differentiate mucinous from non-mucinous cysts with a sensitivity of 98.3%, specificity of 98.9%, and overall accuracy of 98.5% [70]. Furthermore, AI models have shown remarkable potential in IPMNs, with a 2022 study analyzing 3355 EUS images from 43 pancreatectomy patients achieving 99.6% accuracy in distinguishing high from low-grade dysplasia [71]. AI is also being integrated into nCLE imaging, further enhancing diagnostic precision and offering a promising future for automated, high-accuracy evaluation of PCLs.

### 1.3. Role of EUS for Pain Management in Pancreatic Cancer

The pathophysiology of pain in pancreatic cancer is multifactorial, arising from tumor invasion into surrounding nerves, increased intrapancreatic pressure, ischemia, fibrosis, and perineural inflammation. Tumor compression of adjacent structures, pseudocyst formation, and infiltration of the celiac plexus further contribute to debilitating pain, significantly affecting quality of life [72]. EUS-guided pain interventions, particularly celiac plexus block (CPB) and celiac plexus neurolysis (CPN), have emerged as effective alternatives or adjuncts for pain control in pancreatic cancer.

EUS-guided techniques for pain management in pancreatic cancer primarily target the celiac plexus, a major autonomic nerve hub located near the origin of the celiac artery. CPB involves the temporary blockade of pain transmission by injecting a local anesthetic such as bupivacaine, while CPN achieves permanent neurolysis using neurolytic agents like absolute ethanol or phenol. These procedures are performed by advancing a 22-gauge or 19-gauge EUS needle through the gastric wall into the celiac plexus region. Injection can be administered using a unilateral, bilateral, or midline approach, with some studies suggesting that bilateral injections provide superior pain relief and reduce opioid consumption more effectively [73]. An advanced variation, EUS-guided celiac ganglion neurolysis (CGN), specifically targets the individual celiac ganglia rather than the entire plexus, offering a more direct approach. Another novel technique, EUS-guided radiofrequency ablation (EUS-RFA) of the celiac ganglia, uses high-frequency alternating current to thermally ablate nerve tissue, potentially offering more sustained pain relief. EUS-RFA is performed with a 19-gauge, internally cooled RFA needle (e.g., EUSRA; 5–10 mm active tip) connected to a dedicated generator. Under Doppler guidance the tip is seated in a visible celiac ganglion and RF energy is delivered at ~30–50 W for 10–30 s—often stopping when impedance rises toward ~1000 Ω—creating a hyperechoic “bubble” zone; the needle is then withdrawn slightly and energy reapplied to cover the ganglion chain [74,75]. CE-EUS can confirm ablation; key precautions include avoiding intervening vessels and the GI wall (brief pause before withdrawal), with some operators using lower wattage for longer application to promote controlled thermal spread, and giving prophylactic antibiotics along with rectal NSAID [76,77]. Meta-analyses have shown that EUS-CPN provides pain relief in 53–73% of patients within the first 2–4 weeks, with some reporting improvements lasting up to three months [78,79]. EUS-CPN has been shown to improve overall quality of life without significantly altering survival outcomes.

Comparative studies between percutaneous CPN (PQ-CPN) and EUS-CPN have demonstrated comparable efficacy, with EUS-CPN potentially offering better visualization and a lower risk of complications due to its proximity to the gastrointestinal tract [80]. Studies comparing EUS-CPN and EUS-CGN show mixed results. Kamata et al. reported higher complete pain relief with combined EUS-CPN and EUS-CGN (87.8% vs. 60%) but no extension in pain relief duration [81]. However, Levy et al. raised concerns about shorter survival (5.6 vs. 10.5 months) in patients receiving both treatments, despite no difference in opioid use or quality of life [82]. A systematic review by Li et al. confirmed EUS-CGN improved early pain relief but showed variable long-term efficacy [83]. While EUS-CGN may enhance short-term pain control, its long-term impact, especially on survival, remains uncertain, warranting further investigation. Recent studies show clear benefits of EUS-RFA over conventional methods. A randomized trial by Bang et al. compared EUS-RFA to EUS-CPN in 26 patients (14 EUS-CPN, 12 EUS-RFA). EUS-RFA demonstrated superior pain relief, fewer gastrointestinal side effects, and better emotional functioning. Notably, 21% of patients with persistent pain after CPN experienced relief with RFA, highlighting its potential as a rescue therapy [84]. Similarly, Houmani et al. used a novel RFA EUS needle in two patients, both achieving significant pain reduction without adverse events. Unlike EUS-CPN, which has variable and often short-lived efficacy (24–80%), RFA induces permanent ganglion destruction, potentially offering longer-lasting pain relief with more controlled and predictable tissue ablation than ethanol injection [85]. However, higher costs of RFA probes raise concerns about cost-effectiveness. While early findings are promising, more studies are needed to establish long-term efficacy and economic feasibility.

### 1.4. Precision Medicine and Genetic Profiling

Comprehensive genomic profiling (CGP) utilizes NGS to detect actionable mutations and plays a central role in precision medicine by tailoring treatments to individual genetic profiles. PDAC, the most common type of pancreatic cancer, shows frequent mutations in KRAS, TP53, CDKN2A, and SMAD4 (>95% of cases), while less frequent alterations include ATM, BRCA1, ARID1A, and MLL3 [86]. Intraductal papillary mucinous carcinoma (IPMC) often features GNAS and KRAS mutations, with progression marked by TP53 and SMAD4 abnormalities. Pancreatic neuroendocrine carcinomas (PanNECs) are driven by TP53, RB1, and KRAS mutations, contributing to their aggressive behavior [87]. A study analyzing CGP in pancreatic cancer patients found that genetic abnormalities such as BRCA2, BRAF, ERBB2, CDK12, PIK3CA, FGFR2, and EGFR are more commonly observed in patients lacking KRAS mutations. These findings suggest that pancreatic cancer patients without KRAS mutations may particularly benefit from CGP, as it can help identify actionable mutations and expand targeted treatment options. CGP analysis using EUS-FNB has been effective even for low-grade malignancies such as solid-pseudopapillary neoplasms (SPNs), where CTNNB1 mutations were detected in all cases [88].

EUS-FNB has emerged as superior to EUS-FNA for CGP due to higher sample volume, greater tumor cellularity, and better DNA/RNA yields. EUS-FNB specimens have multiple histological tissues and tissue microfragments, that are lacking in EUS-FNA specimens. Kandel et al. reported that the median tumor cellularity of the specimens was 40% and 10%, and the DNA concentration was 5.93 μg/mL and 3.37 μg/mL for EUS-FNB and EUS-FNA, respectively [89]. A minimum of 20% tumor cellularity is recommended for CGP analysis. A study showed that EUS-FNB provides a significantly higher diagnostic adequacy rate (90.9% vs. 66.9% for EUS-FNA, *p* = 0.02) for CGP, multivariate analysis revealed that EUS-FNB (OR: 4.95, 95% CI 1.11–22.05, *p* = 0.04) was the only contributing factor to CGP success [86]. However, it is important to keep in mind that success rate is much higher with surgical samples (90–100% vs. 42–100%), due to better sample volumes and DNA quality [90].

The quality of samples obtained through EUS-FNB is influenced by various factors, including needle characteristics, the number of passes, and sample handling techniques. 19/22G needles have higher CGP success rates compared to 25G needles (60.9% vs. 38.8%, *p* = 0.003) [91]. Notably, EUS-FNB with 19/22G needles achieved 78% fulfillment of CGP requirements with a single puncture compared to 14% with 25G needles [89]. Additionally, Franseen and fork-tip needles used in EUS-FNB demonstrate core tissue acquisition rates exceeding 90% [92]. No concise guidelines exist on number of passes required for an adequate sample, with some studies showing 1–3 punctures of EUS-FNA and FNB can be enough to acquire a good sample [89,93]. It was also observed that fresh tumor tissue yields higher sequencing success (97.4%) than formalin-fixed paraffin-embedded samples (84.8%, *p* < 0.05) [94]. To improve CGP success rates using EUS-FNA/FNB specimens, large-scale studies are needed to optimize needle selection, number of passes, aspiration techniques, and specimen type for adequate tissue yield and quality.

Beyond the selection of needle type, caliber, and tip design, with FNB needles of Franseen or fork-tip geometry currently preferred over FNA, the suction modality adopted during sampling is a key determinant of specimen quality [95]. Available techniques include standard (dry) suction with a 5 to 20 mL syringe, which maximizes specimen quantity at the cost of greater blood contamination; wet suction, in which the needle lumen is pre-flushed with saline to enhance cellularity; the slow-pull (capillary) technique, associated with reduced hemorrhagic artifact and particularly suited to hypervascular lesions; and the no-suction approach [96]. To date, no suction modality has demonstrated clear superiority, and the choice should be tailored to lesion characteristics and operator experience [95]. On-site specimen evaluation further optimizes diagnostic performance. Rapid on-site cytologic evaluation (ROSE) has long been regarded as the reference standard, yet its widespread adoption is limited by the requirement for a dedicated cytopathologist; moreover, with the advent of contemporary FNB needles, its incremental diagnostic benefit appears marginal and may be offset by reduced sample adequacy and prolonged procedure time [96]. Macroscopic on-site evaluation (MOSE), based on direct inspection of the aggregated specimen and considered adequate when a whitish/yellowish core of at least 10 mm is retrieved, has therefore emerged as a pragmatic, operator-driven alternative [96]. In a multicenter randomized non-inferiority trial, Mangiavillano et al. showed that EUS-FNB with MOSE, performed with a 22G Franseen needle, was non-inferior to conventional EUS-FNB with three standard passes in terms of both diagnostic accuracy (90.0% vs. 87.8%) and sample adequacy (93.1% vs. 95.5%), while significantly reducing the median number of passes required to achieve diagnosis (1 vs. 3; *p* < 0.001), with no increase in adverse events [97]. Collectively, these data support an individualized sampling strategy that integrates needle selection, suction modality, and on-site evaluation, preferentially MOSE when ROSE is not available, to maximize diagnostic yield while minimizing procedural burden.

### 1.5. Role of EUS for Pancreatic Cancer Screening

Pancreatic cancer is typically diagnosed at advanced stages, with data showing that only 20% of symptomatic cases are identified when they are resectable or borderline-resectable, while 30% present as locally advanced and 50% as metastatic [98]. Screening aims to detect precursor lesions, such as pancreatic intraepithelial neoplasia-3 (PanIN3) and cystic lesions with high-grade dysplasia, as well as small, localized tumors [99]. Studies demonstrate the value of screening in high-risk populations, with a meta-analysis showing a 1.82% detection rate of pancreatic cancer or high-grade lesions among 1551 high-risk individuals [100]. Additionally, survival outcomes are improved, with median survival reaching 5.3 years for those undergoing surveillance compared to 1.4 years for symptomatic diagnoses [101]. Screen-detected cancers are also more likely to be resectable (90%) and have favorable one- and five-year survival rates of 90% and 60%, respectively [102]. High-risk individuals include those with hereditary cancer syndromes, such as BRCA1 and BRCA2 mutations, which have been linked to an increased risk of pancreatic cancer. Additionally, patients with familial pancreatic cancer (FPC), defined as families with at least two first-degree relatives affected by pancreatic cancer without an identified hereditary syndrome, are at elevated risk, likely due to autosomal-dominant inheritance of a rare allele [103]. ACG and AGA recommend pancreatic cancer screening at age 50 or 10 years younger than the earliest affected family member. Screening starts earlier in high-risk groups, age 40 for PRSS1 and CDKN2A mutation carriers, and age 35 for Peutz–Jeghers syndrome. Non-genetic risk factors, such as tobacco use, chronic pancreatitis, obesity, and new-onset diabetes (especially in individuals over 50 with smoking history or weight loss), increase pancreatic cancer risk, with diabetes raising the risk eightfold and often preceding diagnosis by up to 36 months. In such cases, enhanced diagnostics or shorter surveillance intervals are recommended [99,104].

ASGE recommends both EUS and MRI as primary screening modalities for pancreatic cancer in high-risk individuals. EUS is often preferred as the initial screening tool for patients at very high risk, such as those with Peutz-Jeghers syndrome or Familial Atypical Multiple Mole Melanoma (FAMMM). It is also recommended when EUS can be combined with screening upper endoscopy or colonoscopy, particularly in cases like Lynch syndrome, or when MRI is contraindicated due to conditions such as claustrophobia, contrast allergy, metal implants, or renal failure. Importantly, a linear-array echoendoscope is preferred over a radial one because it has demonstrated a higher detection rate of pancreatic lesions (82% vs. 67%, *p* < 0.001) [103]. On the other hand, MRI is favored for patients at increased risk from anesthesia or for those who prioritize avoiding invasive procedures. MRI may also be advantageous when combined with other imaging techniques, such as enterography for Peutz-Jeghers syndrome [103].

### 1.6. EUS Guided Tumor Ablation for Pancreatic Cancer

EUS-guided tumor ablation has emerged as a promising minimally invasive approach for managing pancreatic cancers, offering alternatives to surgical resection, especially in unresectable or borderline resectable cases. Several EUS-guided ablation techniques have been developed, including RFA, cryothermal ablation, laser ablation, photodynamic therapy (PDT), brachytherapy, high-intensity focused ultrasound (HIFU), and fine-needle injection (FNI) of antitumoral agents. Each method has specific applications depending on the tumor type and clinical scenario.

EUS-RFA, is the most extensively studied and safest among thermal ablation techniques. It uses alternating current and high frequency (460–500 kHz) delivered through monopolar or bipolar electrodes to affected areas, leading to coagulative necrosis, irreversible cell damage, and cell death. In a multicenter study, EUS-RFA achieved complete eradication of cystic pancreatic neoplasms (mean size 28 mm, range 9–60 mm) in 71% of patients, with minimal adverse events such as abdominal pain that resolved within days. For pNETs (mean size 13.1 mm, range 10–20 mm), EUS-RFA has shown an 85.7% complete response rate, according to a 12-patient study, with only one case of pancreatic duct stenosis [105]. Another study further demonstrated 100% complete ablation in insulinoma patients at 12-month follow-up, with no significant complications except with a single case of pancreatic duct stenosis [106]. In PDAC, however, results have been mixed. Morgan et al. demonstrated that the combination of echoendoscopic ethanol ablation with concurrent celiac plexus neurolysis in patients with PDAC was technically feasible, provided effective pain control, and was associated with a favorable safety profile, supporting a role for combined ablation–analgesia approaches in selected unresectable patients [107]. One study achieved a 30% tumor ablation rate with minimal adverse effects, including mild abdominal pain and transient enzyme elevations [108,109]. These findings suggest that while EUS-RFA is effective for cystic neoplasms and functional pNETs, further research is required to optimize its therapeutic role in PDAC, particularly concerning long-term survival outcomes. Randomized studies are also required before EUS-RFA or combined ablation approaches can be recommended routinely for PDAC, particularly with respect to long-term survival outcomes.

EUS-guided PDT enables localized pancreatic ablation without major complications [110]. Laser ablation with the neodymium:yttrium aluminum garnet (Nd:YAG) induces coagulative tumor necrosis and has shown effective, safe pancreatic tissue ablation in animal models, though human data are limited [111]. In a small first-in-human study (*n* = 4), PDT using chlorin e6 achieved 100% technical success, a median ablation volume of 4.0 cm^3^, no complications, and stable disease at ~5 months [112]. Brachytherapy, where radioactive seeds are implanted into the tumor, has shown modest survival benefits. For HIFU, EUS guidance enhances precision, allowing effective tumor ablation and pain reduction, offering potential for both curative and palliative treatments. FNI techniques using agents such as TNFerade (a TNF-alpha gene vector) and ONYX-015 (a replication-selective adenovirus) have also been explored [113]. A study reported promising remission rates post-TNFerade injection, but a Phase III trial failed to confirm survival benefits. Additionally, EUS-FNI has been used for adoptive T-cell therapies, delivering drugs like interferon β-1B directly into tumor sites with notable tumor regression, though larger studies are warranted [114]. Overall, EUS-guided tumor ablation techniques hold significant promise, with varying degrees of success depending on tumor type. EUS-RFA stands out for NETs and cystic neoplasms, while brachytherapy and HIFU show potential for locally advanced PDAC.

Adverse events in EUS-guided ablation mainly result from collateral thermal injury. RFA has been associated with pancreatitis, burns, perforation, and bleeding [115]. Cryotherm shows dose-dependent minor AEs (~43%) in animals, with mild pain, hyperamylasemia, and rare bleeding in humans [116]. Data on PDT and Nd:YAG are limited; small studies report no major complications, though higher laser power may cause thermal injury [117].

## 2. EUS for Cholangiocarcinoma

### 2.1. Diagnosis and Staging of Cholangiocarcinoma

EUS is a critical modality in the diagnosis and staging of cholangiocarcinoma (CCA), offering superior imaging and tissue acquisition capabilities. Studies demonstrate that EUS outperforms CT and MRCP in distinguishing malignant from benign biliary strictures, with reported sensitivities ranging from 25% to 91% and specificities between 89% and 100% [118]. EUS-FNB enhances diagnostic accuracy by providing histological confirmation when imaging alone is inconclusive [119]. For distal CCA, evidence supports the use of standalone EUS in patients without jaundice to prevent the complications of ERCP, which can delay or preclude surgical resection. In patients with jaundice, EUS combined with ERCP allows for comprehensive local assessment, including nodal sampling, cholangiography and intraductal sampling with biopsies and brushing increasing the diagnostic accuracy of combined techniques and exceeding that of either modality alone [120]. EUS accurately assesses tumor size and local invasion, crucial for determining surgical resectability. EUS provides accurate assessment for lymphovascular invasion with sampling potential to evaluate for regional lymph node metastasis, it can identify vascular invasion with an accuracy of up to 85%, which is essential in surgical planning [121].

Adjunctive techniques further enhance EUS utility. EUS-guided biliary drainage (EUS-BD) is increasingly preferred over percutaneous methods when ERCP fails, given its lower morbidity and comparable success rates [122]. For histological diagnosis, linear EUS-FNB outperforms FNA, providing superior tissue cores for molecular analysis, which is vital for targeted therapies. Recent guidelines emphasize the integration of EUS findings into multidisciplinary team discussions, particularly in complex cases like perihilar CCA, where direct cholangioscopy or advanced cytological techniques like FISH may complement EUS for optimal diagnostic yield [120,123].

### 2.2. Role of EUS for Tissue Acquisition

EUS was shown to be more accurate than CT or PET in assessing regional lymph node metastases in CCA patients. However, due to concerns about tumor seeding, some centers exclude patients from liver transplantation after EUS-FNA [124,125]. Current guidelines recommend core needle biopsy for unresectable tumors or metastatic CCAs [126]. Though there is risk of tumor peritoneal seeding when performing EUS-FNA in a transperitoneal manner. A retrospective cohort of 191 patients in a neoadjuvant chemoradiation–to–liver transplantation (LT) protocol (1992–2010) evaluated whether transperitoneal FNA of suspected hilar cholangiocarcinoma increased peritoneal spread. Sixteen patients underwent FNA (13 percutaneous, 3 EUS): cytology was malignant in six, and 5/6 (83%) had peritoneal metastases detected at the pre-transplant staging laparotomy (therefore not after LT). None of the nine patients with negative FNA had peritoneal metastases at staging. Among the 175 patients without any transperitoneal biopsy, peritoneal metastasis at staging occurred in 14/175 (8%), significantly lower than in the FNA-positive subgroup (*p* = 0.0097) [127]. Furthermore, EUS may also aid in detecting early-stage CCA. A study by Sai et al. examined 142 non-icteric patients with elevated alkaline phosphatase and biliary dilation using magnetic resonance cholangiography (MRC). Those with strictures or filling defects underwent EUS; combining MRC with EUS improved sensitivity from 80% to 90% and specificity from 90% to 98%, compared to MRC alone [128]. Another observational prospective study done by Nejad et al., measuring the sensitivity of EUS and EUS-FNA to provide tissue diagnosis, by using surgical pathology as a reference standard. It concluded that EUS detected the tumor in 100% of distal and 83% of proximal CCAs; and the sensitivity of EUS-FNA was significantly higher in distal than in proximal CCA (81% vs. 59%, respectively) [129].

## 3. EUS for Gallbladder Cancer

### 3.1. Diagnosis and Staging of Gallbladder Cancer (GBC)

GBC is a rare and aggressive malignancy of the biliary tract. Approximately 1 in 5 GBC cases in the United States are diagnosed at an early stage, and the median survival for advanced-stage cancer is less than a year [130]. GBC is usually diagnosed incidentally on abdominal imaging. Diagnostic and staging accuracy of CT may be augmented by MRI for accurate assessment of the biliary tree [131].

CEH-EUS improves gallbladder tumor evaluation by assessing tumor vascularity, outperforming B-mode EUS. Polyps ≥ 10 mm with irregular intratumoral vessels or perfusion defects strongly suggest malignancy, with high diagnostic accuracy (sensitivity ~90–93%, specificity ~93–97%) [132,133]. Imazu et al. reported that CEH-EUS showed significantly superior specificity and accuracy to B-EUS in the diagnosis of malignant gallbladder wall thickening (specificity, 98 vs. 65%, respectively; accuracy, 94.4 vs. 73.1%, respectively [134]. Staging remains challenging—CT/MRI detect lymphovascular invasion poorly (~24%), while EUS better assesses nodal disease (sensitivity 81.8%, specificity 92.9%) [135]. EUS-guided tissue acquisition (preferably FNB for molecular profiling) is increasingly important for guiding therapy but is generally reserved for unresectable cases due to seeding risk. CEH-EUS also aids safe targeting during sampling by distinguishing solid tumor from fluid spaces [136].

### 3.2. Role of EUS for Tissue Acquisition

In a study of 187 patients with gallbladder lesions undergoing EUS-FNA/FNB, 18 benign lesions and 169 malignant lesions were identified. Out of the 169 malignant lesions, EUS-FNA and FNB sampled 66 and 28, respectively. Overall sampling adequacy was 98% (184/187). The diagnostic accuracy of EUS-FNA/FNB was 97% (182/187), sensitivity was 97% (164/169), and specificity was 100% (18/18) [137]. A retrospective study by Kang H et al. was done to investigate the efficacy and safety of EUS-FNA/FNB in patients with suspected GBC. It showed that EUS-FNA/FNB was generally safe and exhibited high diagnostic performance for patients with suspected gallbladder cancer [138]. Another retrospective study by Tong T et al. was done to evaluate the efficacy and safety of EUS-FNB in patients with gallbladder masses, concluded that EUS-FNB was a beneficial diagnostic tool for pre-treatment evaluation of patients with gallbladder masses, especially for those who may not be candidates for surgery and need adequate tissue samples to determine the pathological type and guide chemotherapy decisions [139].

## 4. Role of EUS for Metastatic Liver Lesions

### 4.1. Diagnosis and Staging of Metastatic Liver Lesions

Due to the liver’s dual blood supply from the hepatic artery and portal vein, it is the most common site of metastasis for gastrointestinal cancers, and liver metastasis is more common than primary liver cancer [140]. Liver metastasis, regardless of the primary tumor type, usually indicates advanced disease with a poor prognosis [141]. Small-sized metastases are often not detected by CT or MRI. A prospective study by Okasha et al. showed that EUS examination of the liver detected metastases in 16.2%, while CT and MRI detected metastases in 11.2%, making EUS a superior tool for detecting small hepatic lesions [142]. Another study by Awad et al. showed that EUS could detect additional hepatic lesions in 28% of patients with a history of known liver mass that were detected initially by CT scan [143]. A retrospective study by Oh et al. evaluated the effectiveness of CEH-EUS in detecting hepatic metastases and showed that it demonstrated higher sensitivity in detecting small liver metastases (<1 cm) compared to the other modalities stated above [144].

### 4.2. Role of EUS for Tissue Acquisition

A retrospective study conducted by Takano et al. studied both percutaneous biopsy and EUS-FNA and showed that both have equivalent diagnostic qualities for liver tumors, but EUS-FNA was associated with less adverse events [145]. Importantly for metastases, EUS can detect and sample very small deposits—sub-centimeter lesions—particularly in the left lobe; several studies report EUS performance that is comparable for lesions < 2 cm and ≥2 cm, so no firm < 1 cm “failure” cutoff is identified [146,147]. A meta-analysis by Mohan et al. evaluated the efficacy and safety of EUS-guided liver biopsy, reporting a 93.9% success rate for histologic diagnosis with an overall adverse event rate of 2.3% [148]. One large series noted similar diagnostic accuracy for tumors < 20 mm and ≥20 mm, supporting EUS-TA even for small targets [149]. Notably, the adverse event rate with a 19-gauge FNA needle, compared to other core biopsy needles, was lower at 0.9% [148]. A retrospective study by Shuja et al., evaluated the adequacy of EUS-guided liver biopsies in comparison to a transjugular approach. A total of 45% of liver biopsies were performed through EUS-guidance. The study showed that a transjugular approach led to more complications in comparison to EUS-guided procedures (*p* = 0.03) [150]. A study by McCarty et al., compared the efficacy and safety of EUS-guided liver biopsy (EUS-LB), Percutaneous liver biopsy (PCLB), and transjugular liver biopsy (TJLB). Biopsy cumulative adequacy rates for EUS-LB, PCLB, and TJLB were 93.51%, 98.27%, and 97.61%, respectively. But it showed no difference in adverse events between the different approaches [151]. Given its ability to sample small/left-lobe lesions and to combine staging and biopsy in the same endoscopic session, EUS-LB offers a minimally invasive, workflow-efficient alternative when tissue is needed for suspected hepatic metastases.

## 5. Emerging Role of EUS in Biliary and Gastric Outlet Obstruction

### 5.1. Role of EUS for Biliary Obstruction

**Figure 2:**
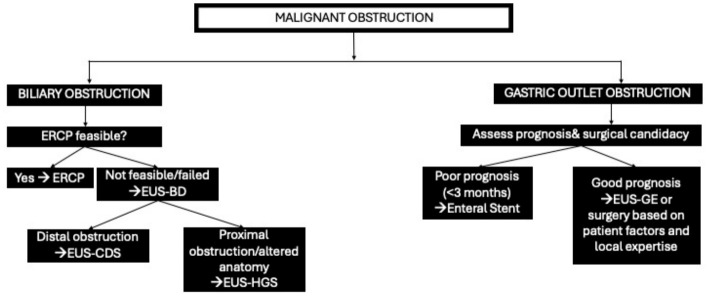
Algorithmic approach to therapeutic endoscopic ultrasound (EUS) in pancreatobiliary malignancies. This schematic outlines the decision-making framework for managing malignant obstruction using EUS-based interventions. In biliary obstruction, endoscopic retrograde cholangiopancreatography (ERCP) remains first-line when feasible; in cases of failure or inaccessibility, EUS-guided biliary drainage (EUS-BD) is performed, with choledochoduodenostomy (EUS-CDS) preferred for distal obstruction and hepaticogastrostomy (EUS-HGS) for proximal obstruction or altered anatomy. In gastric outlet obstruction, management is guided by prognosis, with enteral stenting favored for limited life expectancy and EUS-guided gastroenterostomy (EUS-GE) or surgery for patients with longer expected survival.

EUS-BD is an essential therapeutic modality for the management of biliary obstruction, particularly in cases where ERCP has failed or is contraindicated [152]. The two principal techniques of EUS-BD are EUS-guided hepaticogastrostomy (EUS-HGS) and EUS-guided choledochoduodenostomy (EUS-CDS), which represent complementary transluminal approaches that differ primarily in anatomical access (intrahepatic vs extrahepatic) and clinical indication [153] (Figure 2).

EUS-HGS, first described in 2003, creates a transluminal fistula between the stomach and the left intrahepatic biliary system to achieve internal biliary drainage [154]. Since its initial description, EUS-HGS has evolved substantially with refinements in technique, imaging guidance, and the development of dedicated stents and delivery systems, and it is now regarded as a reliable option for biliary decompression [155]. It is most commonly used when ERCP fails or is not feasible (e.g., duodenal stenosis/gastric outlet obstruction preventing access to the papilla), and increasingly as an alternative approach in selected malignant biliary obstruction scenarios [156]. It is particularly useful when drainage of the left hepatic system is clinically meaningful (e.g., certain hilar patterns where left-sided drainage provides adequate functional decompression, or when the left system cannot be accessed through an indwelling transpapillary self-expandable metal stent (SEMS) [157]. Absolute contraindications generally include significant coagulopathy (e.g., INR > 1.5), severe thrombocytopenia (e.g., <50,000/µL), and hemodynamic instability. Relative contraindications include clinically significant ascites (because it increases stomach–liver separation and bile-leak risk), lack of an adequately dilated left intrahepatic duct (commonly <4–5 mm), an atrophic/nonfunctional left lobe, portal hypertension with perigastric collaterals/varices, left portal vein thrombosis (with collateralization and bleeding risk), and any intervening major vessel or parenchymal lesion along the intended needle tract [158].

EUS-HGS is typically performed from the gastric cardia or proximal body using a linear echoendoscope to identify a dilated left intrahepatic duct, most often segment III (B3) or segment II (B2) [159]. A critical early technical goal is selecting a safe puncture site with careful Doppler interrogation to avoid intervening vasculature and ensuring the puncture trajectory is below the diaphragm to prevent transesophageal puncture. Many operators favor targeting a B3 duct when feasible to reduce transesophageal risk, while recognizing that B2 can offer a straighter course that may facilitate guidewire passage but at the cost of potentially higher mediastinal complication risk if the puncture is too proximal [160]. Once the duct is targeted, a 19G EUS-FNA needle is commonly used (with smaller needles in select minimally dilated ducts), bile is aspirated to confirm intraductal position and decompress the system, and a small amount of diluted contrast is injected under fluoroscopy to delineate ductal anatomy and the level of obstruction [161]. A 0.025-inch guidewire is then advanced, ideally toward the hilum/common hepatic duct (or even across to the contralateral system) and coiled to secure stability for subsequent device exchanges. After guidewire placement, the tract between stomach and duct is created/expanded to allow stent delivery; this can be done with electrocautery (e.g., cystotome) or mechanical/balloon dilation, though current strategies increasingly emphasize minimizing the time between tract creation and stent deployment to reduce bile leak [162,163]. Recent device advances have enabled a “one-step” approach in selected cases, where a fine-gauge stent delivery system permits stent placement without formal tract dilation, aiming to reduce bile leakage during the interval between dilation and deployment [164]. The final step is deployment of a dedicated stent to maintain the hepaticogastric tract; contemporary practice often favors partially covered SEMS designed for HGS, with an uncovered intra-ductal portion to reduce side-branch obstruction and a covered transmural segment to limit bile leak, plus anti-migration features and an adequate intragastric length (commonly several centimeters) to reduce migration into the peritoneum [165].

In terms of effectiveness and safety, multiple series and meta-analyses consistently show high technical success (generally >90%) and clinical efficacy often exceeding 85% for EUS-HGS when performed in appropriate patients and experienced centers [166,167,168]. However, EUS-HGS remains technically demanding and carries a meaningful adverse-event profile. Across studies, overall adverse events have been reported up to roughly 30% in some cohorts, with bile leak among the most common and clinically important complications, alongside cholangitis, bleeding, pneumoperitoneum, stent dysfunction/occlusion, hepatic abscess, and stent migration; fatalities are uncommon but reported [169]. Several factors repeatedly emerge as relevant to safety: ensuring sufficient but not excessive hepatic parenchymal tract (often aiming around 2.5–3 cm to balance tamponade against technical feasibility), avoiding significant ascites or draining it beforehand, minimizing contrast over-injection, and reducing delays between tract manipulation and definitive stent deployment [159]. Notably, data comparing strategies suggest that avoiding tract dilation when technically feasible (using fine-gauge delivery systems) may shorten procedure time and may reduce bile peritonitis rates compared with traditional two-step approaches that include dilation, although device characteristics (pushability, tip design, radial force) and anatomy (duct diameter, wire angle) can influence whether “no-dilation” deployment is possible in a given case [170].

Long-term outcomes are increasingly reported and suggest that recurrent biliary obstruction (RBO) is not rare over time, but many patients maintain clinically useful patency for months. Reported patency estimates vary by cohort and stent type, with examples showing patency around 89% at 30 days, decreasing over time (e.g., 82% at 90 days, 70% at 180 days, and 62% at 1 year in one large series) [166]. Mechanisms of RBO include tumor ingrowth, food impaction/sludge, and hyperplasia at the uncovered portion of the stent; less commonly, bleeding/clot can contribute [158]. Stent selection appears to matter: partially covered SEMS have been associated with lower RBO risk compared with fully covered designs in some analyses, and distal strictures may have more favorable patency in certain datasets [171]. Importantly, when RBO occurs, reintervention through the existing hepaticogastric route is often feasible, using stent-in-stent placement (uncovered SEMS or plastic stents) or endoscopic clearance maneuvers for sludge. EUS-HGS not only a drainage procedure but also a potential long-term access route for repeat biliary therapy in selected patients [172].

EUS-CDS is a transluminal EUS-BD technique that creates a direct communication between the duodenal bulb and the extrahepatic bile duct (CBD) to bypass malignant distal biliary obstruction [173]. Indications are similar as for EUS-HGS. Currently it is increasingly used as a primary drainage strategy even when ERCP is technically feasible, given its direct access route and lack of post-ERCP pancreatitis risk [174]. Candidate selection is largely anatomy-dependent and favors a dilated CBD, with many experts recommending EUS-CDS when the CBD is >12 mm (particularly for highly experienced operators) and >15 mm for non-experts; clinical predictors of adequate dilation may include older age and higher bilirubin in some cohorts [175]. Contraindications include ascites, tumor infiltration of the duodenal bulb, intervening vessels, and situations where safe stent deployment is not possible such as insufficient distal bile duct length below the hilum, anatomic deformation, or interposed structures (e.g., gallbladder/cystic duct along the tract); duodenal stenosis may also increase technical difficulty and is associated with higher failure risk in some analyses [173,174].

From a technical standpoint, EUS-CDS has evolved from early plastic-stent approaches (limited by occlusion and peritonitis risk) to covered SEMS and, since their introduction for EUS-CDS in 2014, widespread adoption of electrocautery-enhanced lumen-apposing metal stents (LAMS) that streamline the procedure [175]. Stepwise, the echoendoscope is positioned in the duodenal bulb to visualize the dilated CBD; the operator confirms an appropriate window (including absence of intervening vasculature on Doppler and avoidance of cystic duct interposition when possible) [176]. Using either a needle-based approach with guidewire placement and subsequent dilation/stenting, or increasingly an all-in-one electrocautery LAMS system, the duodenal wall and bile duct are accessed under EUS guidance, followed by deployment of the distal flange in the bile duct, retraction for firm apposition, and deployment of the proximal flange in the duodenum [176]. Contemporary practice trends toward simplifying tract creation: in large single-center experience spanning technical evolution, early approaches used 22G puncture with contrast, guidewire techniques, and dilation, later transitioning to coaxial electrocautery dilation after 2011, and more recently (since 2020) to dilation-free delivery systems with fine-gauge stent introducers when feasible [177]. Across these eras, procedure-related choices matter: use of a forward-viewing (FV) EUS scope has been associated with improved alignment and may reduce complications like mucosal “double penetration” compared with oblique-viewing scopes, and avoiding high-risk dilation strategies appears to improve safety [177].

Clinically, multiple meta-analyses report high technical success (93.5–96%) and clinical success (88–96%) for EUS-CDS overall, and large real-world series of primary EUS-CDS using covered SEMS demonstrate similarly high technical and clinical success with low early morbidity in experienced hands [178,179]. Adverse events vary by cohort and devices but are generally reported in the 5–20% range, with common events including cholangitis and cholecystitis, and other potential complications such as peritonitis/bilioperitoneum, bleeding, pneumoperitoneum, stent migration, abdominal pain, and double mucosal penetration [180,181,182]. Importantly, safety appears strongly influenced by technique: in one large cohort, early adverse events were substantially higher with needle-knife plus mechanical dilation than with coaxial electrocautery or no dilation, and the use of an oblique-viewing EUS scope emerged as an independent predictor of early adverse events, likely reflecting both inherent alignment limitations and historical-era technique effects [183]. Mispositioned LAMS is a notable procedural complication with a proposed classification system and variable rescue strategies [180].

An increasingly recognized long-term issue after EUS-CDS is stent dysfunction/recurrent biliary obstruction, variably reported from 2.7 to 55% depending on follow-up duration and patient factors [184]. Dysfunction may relate to the covered design and flange geometry (including the perpendicular relationship to the duct wall causing contralateral wall obstruction) and the duodenal bulb location favoring food reflux, leading to sludge/stone or food impaction. Predictors include duodenal invasion in some datasets [180]. Most dysfunction can be managed endoscopically with reintervention strategies such as balloon clearance, and placement of double pigtail stents within the LAMS to maintain lumen orientation and reduce wall apposition; randomized data suggest that adding a pigtail may reduce recurrent biliary obstruction compared with LAMS alone, though reintervention rates may be similar and procedural time is shorter with LAMS alone [185,186].

EUS-CDS and EUS-HGS achieve similarly high rates of technical and clinical success overall, but their relative advantages are context-dependent. A recent prospective study, CABRIOLET_Pro (*n* = 20) showed comparable technical success (100%) for both EUS-CDS and EUS-HGS, with similar clinical success (100% vs. 92.3%). However, EUS-CDS had higher post-procedural adverse events (42.9% vs. 7.7%), mainly severe/fatal cholangitis, and more biliary dysfunctions (71.4% vs. 16.7%), resulting in much shorter dysfunction-free survival (~39 vs. ~268 days), largely due to reflux/food-impaction cholangitis through the choledochoduodenal LAMS [187]. In contrast, a broader meta-analysis by Jiasu Li et al., comparing EUS-CDS and EUS-HGS across indications reported similarly high pooled technical and clinical success (EUS-CDS technical 95.0%, clinical 93.1%; EUS-HGS technical 96.6%, clinical 91.3%), with EUS-CDS demonstrating shorter procedure time and fewer early adverse events overall (12.2% vs. 17.5%; pooled OR for early AEs 0.58) [167]. Taken together, these findings suggest that while EUS-CDS offers procedural efficiency and a favorable early safety profile in general populations, EUS-HGS is the more durable and safer strategy in malignant double obstruction, where duodenobiliary reflux and food impaction can disproportionately compromise EUS-CDS patency and precipitate severe cholangitis.

### 5.2. Role of EUS for Gastric Outlet Obstruction

Endoscopic management has emerged as a minimally invasive alternative to surgical gastroenterostomy (SGE) for both benign and malignant GOO. Available endoscopic options include endoscopic balloon dilation (EBD), enteral stenting (ES) using SEMS or LAMS, and more recently endoscopic ultrasound-guided gastroenterostomy (EUS-GE), which combines the durability of surgical bypass with the minimal invasiveness of endoscopy [188]. EUS-GE is a novel technique that creates a gastroenteric anastomosis under EUS guidance, typically using electrocautery-enhanced lumen-apposing metal stents (EC-LAMS) [189]. While promising, it remains partially standardized and is supported primarily by early-phase studies, pilot cohorts, and case series. Reported technical and clinical success rates exceed 90%, with lower recurrence and reintervention rates compared to duodenal SEMS and long-term patency comparable to surgery [190,191]. Quality-of-life outcomes demonstrate improved oral intake and shorter hospital stays relative to surgical approaches [192].

EUS-GE can be performed via gastrojejunostomy (targeting proximal jejunum) or gastroduodenostomy (third/fourth duodenum), provided bowel loops are within 1 cm of the gastric wall [193]. Modern practice favors EC-LAMS (e.g., Hot Axios, Hot Spaxus, HANAROSTENT Hot Plumber), enabling single-step puncture and deployment. Due to improved dietary tolerance and lower reintervention rates, 20 mm LAMS are preferred over 15 mm stents. Deployment techniques include wireless endoscopic simplified technique (WEST), double-balloon EPASS technique and direct needle puncture technique [194]. EUS-GE is increasingly used in benign GOO refractory to conservative therapy. For longer benign strictures (>5 cm), patients with poor surgical candidacy are better suited for EUS-GE, whereas those with good performance status may undergo SGE [195]. Compared to enteral stenting, meta-analyses show similar technical success but significantly lower reintervention rates [196].

For malignant GOO, treatment selection depends on performance status, prognosis (>2–3 months favors surgery), nutritional status, tumor characteristics, and presence of biliary obstruction. EUS-GE is particularly advantageous in centers with expertise and when long-term patency is desired without surgical morbidity [197]. A recent randomized trial by Bang et al. comparing EUS-GE and SGE demonstrated superiority of EUS-GE, with fewer composite failures (7.9% vs. 38.9%), faster return to solid diet (2 vs. 5 days), shorter hospitalization (3 vs. 9 days), better quality of life, and lower overall costs [198]. Comparative data also suggest lower delayed gastric emptying rates with EUS-GE compared to SGE, with EUS-GE showing promising long-term patency [199,200,201].

## 6. Conclusions

EUS is an indispensable tool in the diagnosis, staging, and tissue sampling of pancreatobiliary diseases (Table 2). Studies have consistently demonstrated its superiority over conventional imaging techniques such as CT, MRI, and MRCP in detecting and characterizing pancreatic and biliary malignancies, differentiating benign from malignant strictures, and guiding therapeutic decision-making. The integration of advanced techniques such as CE-EUS, EUS elastography, and AI has further refined its diagnostic precision. EUS-TA, particularly EUS-FNB, has revolutionized the ability to obtain high-quality histologic samples for cytological, molecular, and genetic analysis. For pancreatic tumors, EUS-FNB has largely replaced EUS-FNA due to its higher diagnostic yield, superior sample adequacy, and ability to facilitate NGS for precision oncology. In CCA and GBC, EUS provides crucial preoperative staging information and enables tissue sampling when ERCP-based techniques are inconclusive. Furthermore, its role in the detection and tissue acquisition of metastatic liver lesions has demonstrated high accuracy and safety, making it a minimally invasive alternative to percutaneous and transjugular approaches. Beyond diagnosis and staging, EUS has become an essential modality for therapeutic interventions, including EUS-BD for malignant biliary obstruction, EUS-guided CPN for pain management in pancreatic cancer, and EUS-guided tumor ablation techniques such as EUS-RFA and photodynamic therapy. These advancements highlight the expanding role of EUS in personalized cancer care, allowing for both local tumor control and systemic treatment stratification. With continuous advancements in technology and technique, EUS remains at the forefront of pancreatobiliary cancer management. Further large-scale studies and innovations in artificial intelligence, molecular profiling, and therapeutic EUS will continue to enhance its utility, improve patient outcomes and shape the future of minimally invasive therapeutic endoscopy.

## Figures and Tables

**Table 1:** This table outlines the endoscopic ultrasound (EUS) characteristics of various pancreatic cystic lesions (PCLs), their preferred imaging mode, and their malignancy potential. Serous cystadenoma (SCN) appears as a microcystic or honeycomb lesion with thin septations and minimal cancer risk. Intraductal papillary mucinous neoplasm (IPMN) shows main pancreatic duct (MPD) dilation or grape-like side-branch cysts, with variable malignancy risk. Mucinous cystic neoplasm (MCN) is unilocular or septated and has moderate cancer potential. Pancreatic neuroendocrine tumors (pNETs) and solid pseudopapillary tumors (SPTs) can be hypervascular or heterogeneous, with some cancer risk. Pancreatic pseudocysts are benign fluid-filled collections without septations or nodules.

Cystic Lesion	EUS Features	Preferred EUS Mode	Malignancy Potential
Serous Cystadenoma (SCN)	Microcystic or honeycomb appearance; thin septations; central scar with calcifications	Fundamental B-mode EUS (FB-EUS)	Very low (~0.01%)
Intraductal Papillary Mucinous Neoplasm (IPMN)	Dilation of main pancreatic duct (MD-IPMN); grape-like cysts (BD-IPMN); mural nodules; ‘fish-eye’ ampulla	Contrast-enhanced harmonic EUS (CH-EUS)	Varies: MD-IPMN (38–68%), BD-IPMN (15–17%)
Mucinous Cystic Neoplasm (MCN)	Unilocular or septated macrocystic cyst; no communication with MPD	Fundamental B-mode EUS (FB-EUS)	Moderate (~10%)
Pancreatic Neuroendocrine Tumor (pNET)	Well-defined, hypervascular; homogenous, may have cystic components	Contrast-enhanced harmonic EUS (CH-EUS)	6–31%
Solid Pseudopapillary Tumor (SPT)	Heterogeneous, hypoechoic; calcifications common	Contrast-enhanced harmonic EUS (CH-EUS)	10%
Pancreatic Pseudocyst	Fluid-filled collection; intracystic debris; no septations or mural nodules	Fundamental B-mode EUS (FB-EUS)	Benign

**Table 2:** Role of EUS in the diagnosis, staging, and tissue sampling of hepatobiliary malignancies. This table summarizes the role of EUS in the evaluation of cholangiocarcinoma, gallbladder carcinoma, and metastatic liver lesions. It highlights EUS’s diagnostic accuracy, its ability to stage malignancies by assessing tumor invasion and lymph node involvement, and its effectiveness in obtaining tissue samples for histopathological confirmation. Supporting evidence from key studies is included, along with potential side effects to consider when utilizing EUS for these malignancies.

Condition	Role of EUS in Diagnosis	Role of EUS in Staging	Role of EUS in Sampling	Evidence Supporting the Same	Adverse Events
Cholangiocarcinoma (CCA)	Superior to CT and MRCP in distinguishing malignant from benign strictures; sensitivity 25–91%, specificity 89–100%.	Assesses tumor size, vascular invasion (accuracy up to 85%), and lymph node metastasis (sensitivity 80–85%, specificity >95%).	EUS-FNA provides histologic confirmation, but concerns exist over tumor seeding, especially for hilar CCA.	Studies show EUS-FNA improves diagnostic accuracy; combined MRC-EUS increases sensitivity from 80% to 90% and specificity from 90% to 98%.	Risk of peritoneal tumor seeding potential complications post-FNA.
Gallbladder Carcinoma (GBC)	CE-EUS improves detection of gallbladder tumors, differentiates malignancy from benign polyps.	EUS-FNA detects regional lymph node involvement with 81.8% sensitivity and 92.9% specificity.	EUS-FNA/FNB has 97% diagnostic accuracy, 97% sensitivity, 100% specificity; CEH-EUS improves accuracy in malignant polyp assessment.	Mitake et al.: EUS detected lymph node metastasis with 89.7% accuracy; Takahashi et al.: EUS-FNA guides chemotherapy selection.	Needle-track seeding concern; EUS-FNA generally reserved for unresectable disease.
Metastatic Liver Lesions	EUS detects smaller hepatic metastases missed by CT/MRI; useful for early detection.	EUS detects additional hepatic lesions in 28% of cases, higher sensitivity for lesions < 1 cm compared to CT/MRI.	EUS-FNA provides equivalent diagnostic yield to percutaneous biopsy but with fewer complications.	Okasha et al.: EUS detected metastases in 16.2% vs. 11.2% by CT/MRI; Mohan et al.: 93.9% histologic success rate with EUS-guided liver biopsy.	Minimal complications with EUS-FNA compared to transjugular/percutaneous biopsy; lower adverse event rate with 19G needles.

## Data Availability

The original contributions presented in this study are included in the article. Further inquiries can be directed to the corresponding author.

## References

[1] Dimagno E.P., Regan P.T., Wilson D.A., Buxton J.L., Hattery R.R., Suarez J.R., Green P.S. (1980). Ultrasonic endoscope. Lancet.

[2] Wiersema M.J., Kochman M.L., Cramer H.M., Tao L.C., Wiersema L.M. (1994). Endosonography-guided real-time fine-needle aspiration biopsy. Gastrointest. Endosc..

[3] Yasuda K., Tanaka Y., Fujimoto S., Nakajima M., Kawai K. (1984). Use of endoscopic ultrasonography in small pancreatic cancer. Scand. J. Gastroenterol. Suppl..

[4] Kaneko M., Katanuma A., Maguchi H., Takahashi K., Osanai M., Yane K., Hashigo S., Harada R., Kato S., Kato R. (2014). Prospective, randomized, comparative study of delineation capability of radial scanning and curved linear array endoscopic ultrasound for the pancreaticobiliary region. Endosc. Int. Open.

[5] Siddiqui U.D., Levy M.J. (2018). EUS-Guided Transluminal Interventions. Gastroenterology.

[6] Stevens T., Parsi M.A. (2010). Endoscopic ultrasound for the diagnosis of chronic pancreatitis. World J. Gastroenterol..

[7] Kanazawa K., Imazu H., Mori N., Ikeda K., Kakutani H., Sumiyama K., Hino S., Ang T.L., Omar S., Tajiri H. (2012). A comparison of electronic radial and curvilinear endoscopic ultrasonography in the detection of pancreatic malignant tumor. Scand. J. Gastroenterol..

[8] Gleeson F.C., Clayton A.C., Zhang L., Clain J.E., Gores G.J., Rajan E., Smyrk T.C., Topazian M.D., Wang K.K., Wiersema M.J. (2008). Adequacy of endoscopic ultrasound core needle biopsy specimen of nonmalignant hepatic parenchymal disease. Clin. Gastroenterol. Hepatol..

[9] Kohut M., Nowak A., Nowakowska-Dulawa E., Marek T., Kaczor R. (2003). Endosonography with linear array instead of endoscopic retrograde cholangiography as the diagnostic tool in patients with moderate suspicion of common bile duct stones. World J. Gastroenterol..

[10] Rocca R., De Angelis C., Castellino F., Masoero G., Daperno M., Sostegni R., Rigazio C., Crocellà L., Lavagna A., Ercole E. (2006). EUS diagnosis and simultaneous endoscopic retrograde cholangiography treatment of common bile duct stones by using an oblique-viewing echoendoscope. Gastrointest. Endosc..

[11] Blackford A.L., Canto M.I., Dbouk M., Hruban R.H., Katona B.W., Chak A., Brand R.E., Syngal S., Farrell J., Kastrinos F. (2024). Pancreatic Cancer Surveillance and Survival of High-Risk Individuals. JAMA Oncol..

[12] American Cancer Society Key Statistics for Pancreatic Cancer. https://www.cancer.org/cancer/pancreatic-cancer/about/key-statistics.html.

[13] Siegel R.L., Miller K.D., Goding Sauer A., Fedewa S.A., Butterly L.F., Anderson J.C., Cercek A., Smith R.A., Jemal A. (2020). Colorectal Cancer statistics, 2020. CA Cancer J. Clin..

[14] Rahib L., Smith B.D., Aizenberg R., Rosenzweig A.B., Fleshman J.M., Matrisian L.M. (2014). Projecting cancer incidence and deaths to 2030: The unexpected burden of thyroid, liver, and pancreas cancers in the United States. Cancer Res..

[15] Degen L., Wiesner W., Beglinger C. (2008). Cystic and solid lesions of the pancreas. Best Pract. Res. Clin. Gastroenterol..

[16] Cloyd J.M., Kopecky K.E., Norton J.A., Kunz P.L., Fisher G.A., Visser B.C., Dua M.M., Park W.G., Poultsides G.A. (2016). Neuroendocrine tumors of the pancreas: Degree of cystic component predicts prognosis. Surgery.

[17] Kosmahl M., Pauser U., Anlauf M., Klöppel G. (2005). Pancreatic ductal adenocarcinomas with cystic features: Neither rare nor uniform. Mod. Pathol..

[18] Machicado J.D., Obuch J.C., Goodman K.A., Schefter T.E., Frakes J., Hoffe S., Latifi K., Simon V.C., Santangelo T., Ezekwe E. (2019). Endoscopic Ultrasound Placement of Preloaded Fiducial Markers Shortens Procedure Time Compared to Back-Loaded Markers. Clin. Gastroenterol. Hepatol..

[19] Chong C.C., Tang R.S., Wong J.C., Chan A.W., Teoh A.Y. (2016). Endoscopic ultrasound of pancreatic lesions. J. Vis. Surg..

[20] Shin C.M., Villa E. (2023). The efficiency of contrast-enhanced endoscopic ultrasound (EUS) combined with EUS elastography for pancreatic cancer diagnosis: A systematic review and meta-analysis. Ultrasonography.

[21] Cui X.-W., Chang J.-M., Kan Q.-C., Chiorean L., Ignee A., Dietrich C.F. (2015). Endoscopic ultrasound elastography: Current status and future perspectives. World J. Gastroenterol..

[22] Lisotti A., Napoleon B., Facciorusso A., Cominardi A., Crinò S.F., Brighi N., Gincul R., Kitano M., Yamashita Y., Marchegiani G. (2021). Contrast-enhanced EUS for the characterization of mural nodules within pancreatic cystic neoplasms: Systematic review and meta-analysis. Gastrointest. Endosc..

[23] Harmsen F.-J., Domagk D., Dietrich C., Hocke M. (2018). Discriminating chronic pancreatitis from pancreatic cancer: Contrast-enhanced EUS and multidetector computed tomography in direct comparison. Endosc. Ultrasound.

[24] Kataoka K., Ishikawa T., Ohno E., Mizutani Y., Iida T., Furukawa K., Nakamura M., Honda T., Ishigami M., Kawashima H. (2022). Differentiation Between Solid Pseudopapillary Neoplasm of the Pancreas and Nonfunctional Pancreatic Neuroendocrine Neoplasm Using Endoscopic Ultrasound. Pancreas.

[25] Humphrey P.E., Alessandrino F., Bellizzi A.M., Mortele K.J. (2015). Non-hyperfunctioning pancreatic endocrine tumors: Multimodality imaging features with histopathological correlation. Abdom. Imaging.

[26] Omoto S., Kitano M., Fukasawa M., Ashida R., Kato H., Shiomi H., Sugimori K., Kanno A., Chiba Y., Takano S. (2021). Tissue harmonic versus contrast-enhanced harmonic endoscopic ultrasonography for the diagnosis of pancreatic tumors: Prospective multicenter study. Dig. Endosc..

[27] Iwasa Y., Iwashita T., Ichikawa H., Mita N., Uemura S., Yoshida K., Iwata K., Mukai T., Yasuda I., Shimizu M. (2021). Efficacy of Contrast-Enhanced Harmonic Endoscopic Ultrasound for Pancreatic Solid Tumors with a Combination of Qualitative and Quantitative Analyses: A Prospective Pilot Study. Dig. Dis. Sci..

[28] Ishikawa R., Kamata K., Hara A., Tanaka H., Okamoto A., Yamazaki T., Nakai A., Omoto S., Minaga K., Yamao K. (2020). Utility of contrast-enhanced harmonic endoscopic ultrasonography fo0r predicting the prognosis of pancreatic neuroendocrine neoplasms. Dig. Endosc..

[29] Constantin A.L., Cazacu I., Burtea D.E., Harbiyeli I.C., Bejinariu N., Popescu C., Serbanescu M., Tabacelia D., Copaescu C., Bhutani M. (2022). Quantitative contrast-enhanced endoscopic ultrasound in pancreatic ductal adenocarcinoma and pancreatic neuroendocrine tumors: Can we predict survival using perfusion parameters? A pilot study. Med. Ultrason..

[30] Shami V.M., Mahajan A., Loch M.M., Stella A.C., Northup P.G., White G.E., Brock A.S., Srinivasan I., De Lange E.E., Kahaleh M. (2011). Comparison between endoscopic ultrasound and magnetic resonance imaging for the staging of pancreatic cancer. Pancreas.

[31] Salom F., Prat F. (2022). Current role of endoscopic ultrasound in the diagnosis and management of pancreatic cancer. World J. Gastrointest. Endosc..

[32] Zhang B., Zhu F., Li P., Yu S., Zhao Y., Li M. (2018). Endoscopic ultrasound elastography in the diagnosis of pancreatic masses: A meta-analysis. Pancreatology.

[33] Dietrich C., Burmeister S., Hollerbach S., Arcidiacono P., Braden B., Fusaroli P., Hocke M., Iglesias-Garcia J., Kitano M., Larghi A. (2020). Do we need elastography for EUS?. Endosc. Ultrasound.

[34] Ying L., Lin X., Xie Z.L., Hu Y.P., Tang K.F., Shi K.Q. (2013). Clinical utility of endoscopic ultrasound elastography for identification of malignant pancreatic masses: A meta-analysis. J. Gastroenterol. Hepatol..

[35] Facciorusso A., Martina M., Buccino R.V., Nacchiero M.C., Muscatiello N. (2018). Diagnostic accuracy of fine-needle aspiration of solid pancreatic lesions guided by endoscopic ultrasound elastography. Ann. Gastroenterol..

[36] Gheorghiu M., Sparchez Z., Rusu I., Bolboacă S.D., Seicean R., Pojoga C., Seicean A. (2022). Direct Comparison of Elastography Endoscopic Ultrasound Fine-Needle Aspiration and B-Mode Endoscopic Ultrasound Fine-Needle Aspiration in Diagnosing Solid Pancreatic Lesions. Int. J. Environ. Res. Public Health.

[37] Ohno E., Kawashima H., Ishikawa T., Iida T., Suzuki H., Uetsuki K., Yashika J., Yamada K., Yoshikawa M., Gibo N. (2020). Diagnostic performance of endoscopic ultrasonography-guided elastography for solid pancreatic lesions: Shear-wave measurements versus strain elastography with histogram analysis. Dig. Endosc..

[38] Otsuka Y., Minaga K., Hara A., Masuta Y., Takenaka M., Chikugo T., Kudo M. (2024). Detective flow imaging endoscopic ultrasound for locating optimal puncture site for a poorly vascularized pancreatic mass. Endosc. Int. Open.

[39] Yamashita Y., Yoshikawa T., Kawaji Y., Tamura T., Hatamaru K., Itonaga M., Ida Y., Maekita T., Iguchi M., Murata S. (2020). Novel endoscopic ultrasonography imaging technique for visualizing microcirculation without contrast enhancement in subepithelial lesions: Prospective study. Dig. Endosc..

[40] Mulqui M.V., Caillol F., Ratone J.P., Hoibian S., Dahel Y., Meunier É., Archimbaud C., Giovannini M. (2024). Detective flow imaging versus contrast-enhanced EUS in solid pancreatic lesions. Endosc. Ultrasound.

[41] Yamashita Y., Yamazaki H., Nakahata A., Shimokawa T., Tamura T., Kawaji Y., Tamura T., Hatamaru K., Itonaga M., Ashida R. (2025). Endoscopic ultrasonography for microvascular imaging without contrast enhancement in the differential diagnosis of pancreatic lesions. Dig. Endosc..

[42] Miwa H., Sugimori K., Yonei S., Yoshimura H., Endo K., Oishi R., Funaoka A., Tsuchiya H., Kaneko T., Numata K. (2024). Differential Diagnosis of Solid Pancreatic Lesions Using Detective Flow Imaging Endoscopic Ultrasonography. Diagnostics.

[43] Oza V.M., Yekula A., Kothari T.H. (2026). Recent Advances in Endoscopic Ultrasound (EUS) for Pancreatic Cystic Lesions. J. Dig. Endosc..

[44] Zhang J., Zhu L., Yao L., Ding X., Chen D., Wu H., Lu Z., Zhou W., Zhang L., An P. (2020). Deep learning–based pancreas segmentation and station recognition system in EUS: Development and validation of a useful training tool (with video). Gastrointest. Endosc..

[45] Wani S., Keswani R.N., Petersen B., Edmundowicz S.A., Walsh C.M., Huang C., Cohen J., Cote G. (2018). Training in EUS and ERCP: Standardizing methods to assess competence. Gastrointest. Endosc..

[46] Krishna S.G., Abdelbaki A., Li Z., Culp S., Xiong X., Napoleon B., Mok S., Bertani H., Feng Y., Kongkam P. (2025). Towards automating risk stratification of intraductal papillary mucinous Neoplasms: Artificial intelligence advances beyond human expertise with confocal laser endomicroscopy. Pancreatology.

[47] Goyal H., Sherazi S.A.A., Gupta S., Perisetti A., Achebe I., Ali A., Tharian B., Thosani N., Sharma N.R. (2022). Application of artificial intelligence in diagnosis of pancreatic malignancies by endoscopic ultrasound: A systemic review. Ther. Adv. Gastroenterol..

[48] Wang X., Tian L., Yu X., Zhang Z., Zhu N., Tang A., Hu S. (2021). Application of a novel artificial intelligence system in guiding the targeted puncture of a pancreatic mass. Endoscopy.

[49] Mohan B.P., Facciorusso A., Khan S.R., Madhu D., Kassab L.L., Ponnada S., Chandan S., Crino S.F., Kochhar G.S., Adler D.G. (2022). Pooled diagnostic parameters of artificial intelligence in EUS image analysis of the pancreas: A descriptive quantitative review. Endosc. Ultrasound.

[50] Litchinko A., Kobayashi K., Halkic N. (2020). A retrospective study of histological outcome for IPMN after surgery in Lausanne, Switzerland: A case series. Ann. Med. Surg..

[51] Springer S., Masica D.L., Dal Molin M., Douville C., Thoburn C.J., Afsari B., Li L., Cohen J.D., Thompson E., Allen P.J. (2019). A multimodality test to guide the management of patients with a pancreatic cyst. Sci. Transl. Med..

[52] Liu Y., Shi S., Hua J., Xu J., Zhang B., Liu J., Yang X.-J., Yu X.-J. (2020). Differentiation of solid-pseudopapillary tumors of the pancreas from pancreatic neuroendocrine tumors by using endoscopic ultrasound. Clin. Res. Hepatol. Gastroenterol..

[53] Zerboni G., Signoretti M., Crippa S., Falconi M., Arcidiacono P.G., Capurso G. (2019). Systematic review and meta-analysis: Prevalence of incidentally detected pancreatic cystic lesions in asymptomatic individuals. Pancreatology.

[54] Keane M.G., Shamali A., Nilsson L.N., Antila A., Millastre Bocos J., Marijinissen Van Zanten M., Verdejo Gil C., Maisonneuve P., Vaalavuo Y., Hoskins T. (2018). Risk of malignancy in resected pancreatic mucinous cystic neoplasms. BJS.

[55] Jais B., Rebours V., Malleo G., Salvia R., Fontana M., Maggino L., Bassi C., Manfredi R., Moran R., Lennon A.M. (2015). Serous cystic neoplasm of the pancreas: A multinational study of 2622 patients under the auspices of the International Association of Pancreatology and European Pancreatic Club (European Study Group on Cystic Tumors of the Pancreas). Gut.

[56] Rangwani S., Juakiem W., Krishna S.G., El-Dika S. (2023). Role of Endoscopic Ultrasound in the Evaluation of Pancreatic Cystic Neoplasms: A Concise Review. Diagnostics.

[57] Tanaka M., Fernández-del Castillo C., Kamisawa T., Jang J.Y., Levy P., Ohtsuka T., Salvia R., Shimizu Y., Tada M., Wolfgang C.L. (2017). Revisions of international consensus Fukuoka guidelines for the management of IPMN of the pancreas. Pancreatology.

[58] Anand N., Sampath K., Wu B.U. (2013). Cyst features and risk of malignancy in intraductal papillary mucinous neoplasms of the pancreas: A meta-analysis. Clin. Gastroenterol. Hepatol..

[59] Marchegiani G., Andrianello S., Borin A., Dal Borgo C., Perri G., Pollini T., Romanò G., D’Onofrio M., Gabbrielli A., Scarpa A. (2018). Systematic review, meta-analysis, and a high-volume center experience supporting the new role of mural nodules proposed by the updated 2017 international guidelines on IPMN of the pancreas. Surgery.

[60] Iwaya H., Hijioka S., Mizuno N., Kuwahara T., Okuno N., Tajika M., Tanaka T., Ishihara M., Hirayama Y., Onishi S. (2019). Usefulness of septal thickness measurement on endoscopic ultrasound as a predictor of malignancy of branched-duct and mixed-type intraductal papillary mucinous neoplasm of the pancreas. Dig. Endosc..

[61] Postlewait L.M., Ethun C.G., McInnis M.R., Merchant N., Parikh A., Idrees K., Isom C.A., Hawkins W., Fields R.C., Strand M. (2017). Association of Preoperative Risk Factors With Malignancy in Pancreatic Mucinous Cystic Neoplasms. JAMA Surg..

[62] Olar M.P., Bolboacă S.D., Pojoga C., Moșteanu O., Gheorghiu M., Seicean R., Rusu I., Sparchez Z., Al Hajjar N., Seicean A. (2022). Clinical Utility of the Contrast-Enhanced Endoscopic Ultrasound Guided Fine Needle Aspiration in the Diagnosis of Pancreatic Cyst. Diagnostics.

[63] (2018). The European Study Group on Cystic Tumours of the Pancreas. European evidence-based guidelines on pancreatic cystic neoplasms. Gut.

[64] Smith Z.L., Satyavada S., Simons-Linares R., Mok S.R., Moreno B.M., Aparicio J.R., Chahal P. (2021). Intracystic Glucose and Carcinoembryonic Antigen in Differentiating Histologically Confirmed Pancreatic Mucinous Neoplastic Cysts. Am. J. Gastroenterol..

[65] McCarty T.R., Paleti S., Rustagi T. (2021). Molecular analysis of EUS-acquired pancreatic cyst fluid for KRAS and GNAS mutations for diagnosis of intraductal papillary mucinous neoplasia and mucinous cystic lesions: A systematic review and meta-analysis. Gastrointest. Endosc..

[66] Paniccia A., Polanco P.M., Boone B.A., Wald A.I., McGrath K., Brand R.E., Khalid A., Kubiliun N., O’BRoin-Lennon A.M., Park W.G. (2022). Prospective, Multi-Institutional, Real-Time Next-Generation Sequencing of Pancreatic Cyst Fluid Reveals Diverse Genomic Alterations That Improve the Clinical Management of Pancreatic Cysts. Gastroenterology.

[67] Robles-Medranda C., Olmos J.I., Puga-Tejada M., Oleas R., Baquerizo-Burgos J., Arevalo-Mora M., Zavala R.D.V., Nebel J.A., Loffredo D.C., Pitanga-Lukashok H. (2022). Endoscopic ultrasound-guided through-the-needle microforceps biopsy and needle-based confocal laser-endomicroscopy increase detection of potentially malignant pancreatic cystic lesions: A single-center study. World J. Gastrointest. Endosc..

[68] Kovacevic B., Antonelli G., Klausen P., Hassan C., Larghi A., Vilmann P., Karstensen J. (2021). EUS-guided biopsy versus confocal laser endomicroscopy in patients with pancreatic cystic lesions: A systematic review and meta-analysis. Endosc. Ultrasound.

[69] Krishna S.G., Hart P.A., DeWitt J.M., DiMaio C.J., Kongkam P., Napoleon B., Othman M.O., Tan D.M.Y., Strobel S.G., Stanich P.P. (2020). EUS-guided confocal laser endomicroscopy: Prediction of dysplasia in intraductal papillary mucinous neoplasms (with video). Gastrointest. Endosc..

[70] Vilas-Boas F., Ribeiro T., Afonso J., Cardoso H., Lopes S., Moutinho-Ribeiro P., Ferreira J., Mascarenhas-Saraiva M., Macedo G. (2022). Deep Learning for Automatic Differentiation of Mucinous versus Non-Mucinous Pancreatic Cystic Lesions: A Pilot Study. Diagnostics.

[71] Schulz D.A.H.O., Heilmaier M., Phillip V., Treiber M., Mayr U., Lahmer T., Mueller J., Demir I.E., Friess H., Reichert M. (2022). Accurate prediction of histological grading of intraductal papillary mucinous neoplasia using deep learning. Endoscopy.

[72] Minaga K., Takenaka M., Kamata K., Yoshikawa T., Nakai A., Omoto S., Miyata T., Yamao K., Imai H., Sakamoto H. (2018). Alleviating Pancreatic Cancer-Associated Pain Using Endoscopic Ultrasound-Guided Neurolysis. Cancers.

[73] Sun S., Sahai A.V., Wyse J.M., Battat R., Saftoiu A., Siddiqui A., Leong A.T., Arias B.L.A., Fabbri C., Adler D.G. (2017). Practice guidelines for endoscopic ultrasound-guided celiac plexus neurolysis. Endosc. Ultrasound.

[74] Xu R., Zhang K., Ge N., Sun S. (2024). EUS-guided interventional therapies for pancreatic diseases. Front. Med..

[75] DeWitt J.M., Sandrasegaran K., O’NEil B., House M.G., Zyromski N.J., Sehdev A., Perkins S.M., Flynn J., McCranor L., Shahda S. (2019). Phase 1 study of EUS-guided photodynamic therapy for locally advanced pancreatic cancer. Gastrointest. Endosc..

[76] Rimbaș M., Dumitru A.-C., Tripodi G., Larghi A. (2024). EUS-Guided Radiofrequency Ablation Therapy for Pancreatic Neoplasia. Diagnostics.

[77] Yousaf M.N., Ehsan H., Muneeb A., Wahab A., Sana M.K., Neupane K., Chaudhary F.S. (2021). Role of Radiofrequency Ablation in the Management of Unresectable Pancreatic Cancer. Front. Med..

[78] Koulouris A., Alexandre L., Hart A., Clark A. (2021). Endoscopic ultrasound-guided celiac plexus neurolysis (EUS-CPN) technique and analgesic efficacy in patients with pancreatic cancer: A systematic review and meta-analysis. Pancreatology.

[79] Asif A.A., Walayat S.K., Bechtold M.L., Revanur V., Puli S.R. (2021). EUS-guided celiac plexus neurolysis for pain in pancreatic cancer patients—A meta-analysis and systematic review. J. Community Hosp. Intern. Med. Perspect..

[80] Yoon W.J., Oh Y., Yoo C., Jang S., Cho S.-S., Suh J.-H., Choi S.-S., Park D.H. (2020). EUS-Guided Versus Percutaneous Celiac Neurolysis for the Management of Intractable Pain Due to Unresectable Pancreatic Cancer: A Randomized Clinical Trial. J. Clin. Med..

[81] Kamata K., Kinoshita M., Kinoshita I., Imai H., Ogura T., Matsumoto H., Minaga K., Chiba Y., Takenaka M., Kudo M. (2022). Efficacy of EUS-guided celiac plexus neurolysis in combination with EUS-guided celiac ganglia neurolysis for pancreatic cancer-associated pain: A multicenter prospective trial. Int. J. Clin. Oncol..

[82] Levy M.J., Gleeson F.C., Topazian M.D., Fujii-Lau L.L., Enders F.T., Larson J.J., Mara K., Abu Dayyeh B.K., Alberts S.R., Hallemeier C.L. (2019). Combined Celiac Ganglia and Plexus Neurolysis Shortens Survival, Without Benefit, vs Plexus Neurolysis Alone. Clin. Gastroenterol. Hepatol..

[83] Li M., Wang Z., Chen Y., Wu Z., Huang X., Wu C., Tian B. (2021). EUS-CGN versus EUS-CPN in pancreatic cancer: A qualitative systematic review. Medicine.

[84] Bang J.Y., Sutton B., Hawes R.H., Varadarajulu S. (2019). EUS-guided celiac ganglion radiofrequency ablation versus celiac plexus neurolysis for palliation of pain in pancreatic cancer: A randomized controlled trial (with videos). Gastrointest. Endosc..

[85] Houmani Z.S., Noureddine M.S. (2020). EUS-guided celiac plexus radiofrequency ablation using a novel device. VideoGIE.

[86] Elhanafi S., Mahmud N., Vergara N., Kochman M.L., Das K.K., Ginsberg G.G., Rajala M., Chandrasekhara V. (2018). Comparison of endoscopic ultrasound tissue acquisition methods for genomic analysis of pancreatic cancer. J. Gastroenterol. Hepatol..

[87] Karamitopoulou E. (2022). Molecular Pathology of Pancreatic Cancer. Cancers.

[88] Umemoto K., Yamamoto H., Oikawa R., Takeda H., Doi A., Horie Y., Arai H., Ogura T., Mizukami T., Izawa N. (2022). The Molecular Landscape of Pancreatobiliary Cancers for Novel Targeted Therapies From Real-World Genomic Profiling. JNCI J. Natl. Cancer Inst..

[89] Kandel P., Nassar A., Gomez V., Raimondo M., Woodward T.A., Crook J.E., Fares N.S., Wallace M.B. (2020). Comparison of endoscopic ultrasound-guided fine-needle biopsy versus fine-needle aspiration for genomic profiling and DNA yield in pancreatic cancer: A randomized crossover trial. Endoscopy.

[90] Kondo T., Matsubara J., Quy P.N., Fukuyama K., Nomura M., Funakoshi T., Doi K., Sakamori Y., Yoshioka M., Yokoyama A. (2020). Comprehensive genomic profiling for patients with chemotherapy-naïve advanced cancer. Cancer Sci..

[91] Park J.K., Lee J.H., Noh D.H., Park J.K., Lee K.T., Lee J.K., Lee K.H., Jang K.T., Cho J. (2019). Factors of Endoscopic Ultrasound-Guided Tissue Acquisition for Successful Next-Generation Sequencing in Pancreatic Ductal Adenocarcinoma. Gut Liver.

[92] Facciorusso A., Del Prete V., Buccino V.R., Purohit P., Setia P., Muscatiello N. (2019). Diagnostic yield of Franseen and Fork-Tip biopsy needles for endoscopic ultrasound-guided tissue acquisition: A meta-analysis. Endosc. Int. Open.

[93] Asokkumar R., Ka C.Y., Loh T., Ling L.K., San T.G., Ying H., Tan D., Khor C., Lim T., Soetikno R. (2019). Comparison of tissue and molecular yield between fine-needle biopsy (FNB) and fine-needle aspiration (FNA): A randomized study. Endosc. Int. Open.

[94] Eso Y., Kou T., Nagai H., Kim Y.H., Kanai M., Matsumoto S., Mishima M., Arasawa S., Iguchi E., Nakamura F. (2019). Utility of ultrasound-guided liver tumor biopsy for next-generation sequencing-based clinical sequencing. Hepatol. Res..

[95] Facciorusso A., Crinò S.F., Gkolfakis P., Spadaccini M., Arvanitakis M., Beyna T., Bronswijk M., Dhar J., Ellrichmann M., Gincul R. (2025). Diagnostic work-up of bile duct strictures: European Society of Gastrointestinal Endoscopy (ESGE) Guideline. Endoscopy.

[96] Machicado J.D., Sheth S.G., Chalhoub J.M., Forbes N., Desai M., Ngamruengphong S., Papachristou G.I., Sahai V., Nassour I., Abidi W. (2024). American Society for Gastrointestinal Endoscopy guideline on role of endoscopy in the diagnosis and management of solid pancreatic masses: Methodology and review of evidence. Gastrointest. Endosc..

[97] Mangiavillano B., Crinò S.F., Facciorusso A., Di Matteo F., Barbera C., Larghi A., Rizzatti G., Carrara S., Spadaccini M., Auriemma F. (2022). Endoscopic ultrasound-guided fine-needle biopsy with or without macroscopic on-site evaluation: A randomized controlled noninferiority trial. Endoscopy.

[98] Henrikson N.B., Aiello Bowles E.J., Blasi P.R., Morrison C.C., Nguyen M., Pillarisetty V.G., Lin J.S. (2019). Screening for Pancreatic Cancer: Updated Evidence Report and Systematic Review for the US Preventive Services Task Force. JAMA.

[99] Aslanian H.R., Lee J.H., Canto M.I. (2020). AGA Clinical Practice Update on Pancreas Cancer Screening in High-Risk Individuals: Expert Review. Gastroenterology.

[100] Paiella S., Salvia R., De Pastena M., Pollini T., Casetti L., Landoni L., Esposito A., Marchegiani G., Malleo G., De Marchi G. (2018). Screening/surveillance programs for pancreatic cancer in familial high-risk individuals: A systematic review and proportion meta-analysis of screening results. Pancreatology.

[101] Canto M.I., Almario J.A., Schulick R.D., Yeo C.J., Klein A., Blackford A., Shin E.J., Sanyal A., Yenokyan G., Lennon A.M. (2018). Risk of Neoplastic Progression in Individuals at High Risk for Pancreatic Cancer Undergoing Long-term Surveillance. Gastroenterology.

[102] Canto M.I., Kerdsirichairat T., Yeo C.J., Hruban R.H., Shin E.J., Almario J.A., Blackford A., Ford M., Klein A.P., Javed A.A. (2020). Surgical Outcomes After Pancreatic Resection of Screening-Detected Lesions in Individuals at High Risk for Developing Pancreatic Cancer. J. Gastrointest. Surg..

[103] Sawhney M.S., Calderwood A.H., Thosani N.C., Rebbeck T.R., Wani S., Canto M.I., Fishman D.S., Golan T., Hidalgo M., Kwon R.S. (2022). ASGE guideline on screening for pancreatic cancer in individuals with genetic susceptibility: Summary and recommendations. Gastrointest. Endosc..

[104] Syngal S., Brand R.E., Church J.M., Giardiello F.M., Hampel H.L., Burt R.W. (2015). ACG Clinical Guideline: Genetic Testing and Management of Hereditary Gastrointestinal Cancer Syndromes. Am. J. Gastroenterol..

[105] Barthet M., Giovannini M., Lesavre N., Boustiere C., Napoleon B., Koch S., Gasmi M., Vanbiervliet G., Gonzalez J.-M. (2019). Endoscopic ultrasound-guided radiofrequency ablation for pancreatic neuroendocrine tumors and pancreatic cystic neoplasms: A prospective multicenter study. Endoscopy.

[106] de Nucci G., Imperatore N., Mandelli E.D., di Nuovo F., D’uRbano C., Manes G. (2020). Endoscopic ultrasound-guided radiofrequency ablation of pancreatic neuroendocrine tumors: A case series. Endosc. Int. Open.

[107] Morgan A.D., Ramai D., Bandaru P., Crino S.F., Facciorusso A. (2023). Endoscopic Ultrasound-Guided Therapies in Patients with Pancreatic Neuroendocrine Tumors. Endocr. Metab. Immune Disord.-Drug Targets.

[108] Wang J., Wang Y., Zhao Y., Wu X., Zhang M., Hou W., Chen Q., Cheng B. (2021). Endoscopic ultrasound-guided radiofrequency ablation of unresectable pancreatic cancer with low ablation power and multiple applications: A preliminary study of 11 patients. Ann. Palliat. Med..

[109] Crinò S.F., D’oNofrio M., Bernardoni L., Frulloni L., Iannelli M., Malleo G., Paiella S., Larghi A., Gabbrielli A. (2018). EUS-guided Radiofrequency Ablation (EUS-RFA) of Solid Pancreatic Neoplasm Using an 18-gauge Needle Electrode: Feasibility, Safety, and Technical Success. J. Gastrointest. Liver Dis..

[110] Chan H.H., Nishioka N.S., Mino M., Lauwers G.Y., Puricelli W.P., Collier K.N., Brugge W.R. (2004). EUS-guided photodynamic therapy of the pancreas: A pilot study. Gastrointest. Endosc..

[111] Di Matteo F., Martino M., Rea R., Pandolfi M., Panzera F., Stigliano E., Schena E., Saccomandi P., Silvestri S., Pacella C.M. (2013). US-guided application of Nd:YAG laser in porcine pancreatic tissue: An ex vivo study and numerical simulation. Gastrointest. Endosc..

[112] Choi J.-H., Oh D., Lee J.H., Park J.-H., Kim K.-P., Lee S.S., Lee Y.-J., Lim Y.-S., Song T.J., Seo D.-W. (2015). Initial human experience of endoscopic ultrasound-guided photodynamic therapy with a novel photosensitizer and a flexible laser-light catheter. Endoscopy.

[113] Alvarez-Sánchez M.-V., Napoléon B. (2016). Review of endoscopic radiofrequency in biliopancreatic tumours with emphasis on clinical benefits, controversies and safety. World J. Gastroenterol..

[114] Lakhtakia S., Seo D. (2017). Endoscopic ultrasonography-guided tumor ablation. Dig. Endosc..

[115] Signoretti M., Valente R., Repici A., Fave G.D., Capurso G., Carrara S. (2017). Endoscopy-guided ablation of pancreatic lesions: Technical possibilities and clinical outlook. World J. Gastrointest. Endosc..

[116] Khorana A.A., Mangu P.B., Katz M.H. (2017). Potentially Curable Pancreatic Cancer: American Society of Clinical Oncology Clinical Practice Guideline Update Summary. J. Oncol. Pract..

[117] Dabizzi E., Arcidiacono P.G. (2017). EUS-guided solid pancreatic tumor ablation. Endosc. Ultrasound.

[118] Navaneethan U., Njei B., Venkatesh P.G., Lourdusamy V., Sanaka M.R. (2014). Endoscopic ultrasound in the diagnosis of cholangiocarcinoma as the etiology of biliary strictures: A systematic review and meta-analysis. Gastroenterol. Rep..

[119] Shin D.W., Moon S.-H., Kim J.H. (2023). Diagnosis of Cholangiocarcinoma. Diagnostics.

[120] Rushbrook S.M., Kendall T.J., Zen Y., Albazaz R., Manoharan P., Pereira S.P., Sturgess R., Davidson B.R., Malik H.Z., Manas D. (2023). British Society of Gastroenterology guidelines for the diagnosis and management of cholangiocarcinoma. Gut.

[121] Krampitz G.W., Aloia T.A. (2019). Staging of biliary and primary liver tumors: Current recommendations and workup. Surg. Oncol. Clin..

[122] Sadeghi A., Mohamadnejad M., Islami F., Keshtkar A., Biglari M., Malekzadeh R., Eloubeidi M.A. (2016). Diagnostic yield of EUS-guided FNA for malignant biliary stricture: A systematic review and meta-analysis. Gastrointest. Endosc..

[123] de Moura D.T.H., Ryou M., de Moura E.G.H., Ribeiro I.B., Bernardo W.M., Thompson C.C. (2020). Endoscopic Ultrasound-Guided Fine Needle Aspiration and Endoscopic Retrograde Cholangiopancreatography-Based Tissue Sampling in Suspected Malignant Biliary Strictures: A Meta-Analysis of Same-Session Procedures. Clin. Endosc..

[124] Anderson M.A., Appalaneni V., Ben-Menachem T., Decker G.A., Early D.S., Evans J.A., Fanelli R.D., Fisher D.A., Fisher L.R., American Society for Gastrointestinal Endoscopy (ASGE) Standards of Practice Committee (2013). The role of endoscopy in the evaluation and treatment of patients with biliary neoplasia. Gastrointest. Endosc..

[125] Meara R.S., Jhala D., Eloubeidi M.A., Eltoum I., Chhieng D.C., Crowe D.R., Varadarajulu S., Jhala N. (2006). Endoscopic ultrasound-guided FNA biopsy of bile duct and gallbladder: Analysis of 53 cases. Cytopathology.

[126] Bowlus C.L., Arrivé L., Bergquist A., Deneau M., Forman L., Ilyas S.I., Lunsford K.E., Martinez M., Sapisochin G., Shroff R. (2022). AASLD practice guidance on primary sclerosing cholangitis and cholangiocarcinoma. Hepatology.

[127] Heimbach J.K., Sanchez W., Rosen C.B., Gores G.J. (2011). Trans-peritoneal fine needle aspiration biopsy of hilar cholangiocarcinoma is associated with disease dissemination. HPB.

[128] Sai J.K., Suyama M., Kubokawa Y., Watanabe S., Maehara T. (2009). Early detection of extrahepatic bile-duct carcinomas in the nonicteric stage by using MRCP followed by EUS. Gastrointest. Endosc..

[129] Mohamadnejad M., DeWitt J.M., Sherman S., LeBlanc J.K., Pitt H.A., House M.G., Jones K.J., Fogel E.L., McHenry L., Watkins J.L. (2011). Role of EUS for preoperative evaluation of cholangiocarcinoma: A large single-center experience. Gastrointest. Endosc..

[130] Rawla P., Sunkara T., Thandra K.C., Barsouk A. (2019). Epidemiology of gallbladder cancer. Clin. Exp. Hepatol..

[131] Hijioka S., Nagashio Y., Ohba A., Maruki Y., Okusaka T. (2021). The Role of EUS and EUS-FNA in Differentiating Benign and Malignant Gallbladder Lesions. Diagnostics.

[132] Tamura T., Ashida R., Kitano M. (2022). The usefulness of endoscopic ultrasound in the diagnosis of gallbladder lesions. Front. Med..

[133] Choi J.H., Seo D.W., Choi J.H., Park D.H., Lee S.S., Lee S.K., Kim M.H. (2013). Utility of contrast-enhanced harmonic EUS in the diagnosis of malignant gallbladder polyps (with videos). Gastrointest. Endosc..

[134] Imazu H., Mori N., Kanazawa K., Chiba M., Toyoizumi H., Torisu Y., Koyama S., Hino S., Ang T.L., Tajiri H. (2014). Contrast-enhanced harmonic endoscopic ultrasonography in the differential diagnosis of gallbladder wall thickening. Dig. Dis. Sci..

[135] Kokudo N., Makuuchi M., Natori T., Sakamoto Y., Yamamoto J., Seki M., Noie T., Sugawara Y., Imamura H., Asahara S. (2003). Strategies for surgical treatment of gallbladder carcinoma based on information available before resection. Arch. Surg..

[136] Takahashi K., Ozawa E., Shimakura A., Mori T., Miyaaki H., Nakao K. (2024). Recent Advances in Endoscopic Ultrasound for Gallbladder Disease Diagnosis. Diagnostics.

[137] Kuraishi Y., Hara K., Haba S., Kuwahara T., Okuno N., Yanaidani T., Ishikawa S., Yasuda T., Yamada M., Fukui T. (2023). Diagnostic performance and safety of endoscopic ultrasound-guided fine-needle aspiration/biopsy for gallbladder lesions. Dig. Endosc..

[138] Kang H., Kim S.J., Do M.Y., Kim E.J., Kim Y.S., Jang S.I., Bang S., Cho J.H. (2024). EUS-guided FNA and biopsy for cytohistologic diagnosis of gallbladder cancer: A multicenter retrospective study. Gastrointest. Endosc..

[139] Kanthan R., Senger J.-L., Ahmed S., Kanthan S.C. (2015). Gallbladder Cancer in the 21st Century. J. Oncol..

[140] Weiss L. (1992). Comments on hematogenous metastatic patterns in humans as revealed by autopsy. Clin. Exp. Metastasis.

[141] Siegel R.L., Miller K.D., Fuchs H.E., Jemal A. (2021). Cancer Statistics, 2021. CA Cancer J. Clin..

[142] Okasha H., Wifi M.-N., Awad A., Abdelfatah Y., Abdelfatah D., El-Sawy S., Alzamzamy A., Abou-Elenin S., Abou-Elmagd A., ElHusseiny R. (2021). Role of EUS in detection of liver metastasis not seen by computed tomography or magnetic resonance imaging during staging of pancreatic, gastrointestinal, and thoracic malignancies. Endosc. Ultrasound.

[143] Awad S.S., Fagan S., Abudayyeh S., Karim N., Berger D.H., Ayub K. (2002). Preoperative evaluation of hepatic lesions for the staging of hepatocellular and metastatic liver carcinoma using endoscopic ultrasonography. Am. J. Surg..

[144] Seo D.-W., Oh D., Hong S.-M., Song T.J., Park D.H., Lee S.S., Lee S.K., Kim M.-H. (2017). Endoscopic ultrasound-guided fine-needle aspiration can target right liver mass. Endosc. Ultrasound.

[145] Takano Y., Noda J., Yamawaki M., Azami T., Kobayashi T., Niiya F., Maruoka N., Norose T., Ohike N., Wakabayashi T. (2021). Comparative Study of an Ultrasound-guided Percutaneous Biopsy and Endoscopic Ultrasound-guided Fine-needle Aspiration for Liver Tumors. Intern. Med..

[146] Ichim V.A., Chira R.I., Mircea P.A. (2019). Diagnostic yield of endoscopic ultrasound-guided biopsy of focal liver lesions. Med. Pharm. Rep..

[147] Zhang L., Cai Z., Rodriguez J., Zhang S., Thomas J., Zhu H. (2020). Fine needle biopsy of malignant tumors of the liver: A retrospective study of 624 cases from a single institution experience. Diagn. Pathol..

[148] Mohan B.P., Shakhatreh M., Garg R., Ponnada S., Adler D.G. (2019). Efficacy and safety of EUS-guided liver biopsy: A systematic review and meta-analysis. Gastrointest. Endosc..

[149] Chen Q.-W., Cheng C.-S., Chen H., Ning Z.-Y., Tang S.-F., Zhang X., Zhu X.-Y., Vargulick S., Shen Y.-H., Hua Y.-Q. (2014). Effectiveness and complications of ultrasound guided fine needle aspiration for primary liver cancer in a Chinese population with serum α-fetoprotein levels≤ 200 ng/ml-a study based on 4,312 patients. PLoS ONE.

[150] Shuja A., Alkhasawneh A., Fialho A., Fialho A., Shukri A., Harris C., Smotherman C., Malespin M., De Melo S.W. (2019). Comparison of EUS-guided versus percutaneous and transjugular approaches for the performance of liver biopsies. Dig. Liver Dis..

[151] McCarty T.R., Bazarbashi A.N., Njei B., Ryou M., Aslanian H.R., Muniraj T. (2020). Endoscopic Ultrasound-Guided, Percutaneous, and Transjugular Liver Biopsy: A Comparative Systematic Review and Meta-Analysis. Clin. Endosc..

[152] Dumonceau J.-M., Tringali A., Papanikolaou I.S., Blero D., Mangiavillano B., Schmidt A., Vanbiervliet G., Costamagna G., Devière J., García-Cano J. (2018). Endoscopic biliary stenting: Indications, choice of stents, and results: European Society of Gastrointestinal Endoscopy (ESGE) Clinical Guideline—Updated October 2017. Endoscopy.

[153] Lambin T., Leblanc S., Napoléon B. (2025). Advances in EUS-Guided Biliary Drainage for the Management of Pancreatic Cancer. Cancers.

[154] Paik W.H., Lee T.H., Park D.H., Choi J.H., Kim S.O., Jang S., Kim D.U., Shim J.H., Song T.J., Lee S.S. (2018). EUS-guided biliary drainage versus ERCP for the primary palliation of malignant biliary obstruction: A multicenter randomized clinical trial. Am. J. Gastroenterol..

[155] Ginnaram S.R., Nugooru S., Tahir D., Devine K., Shaikh A.R., Yarra P., Walter J. (2024). Comparative efficacy of endoscopic ultrasound-guided biliary drainage versus endoscopic retrograde cholangiopancreatography as first-line palliation in malignant distal biliary obstruction: A systematic review and meta-analysis. Ann. Gastroenterol..

[156] Itoi T., Yamamoto K., Tsuchiya T., Tanaka R., Tonozuka R., Honjo M., Mukai S., Fujita M., Asai Y., Matsunami Y. (2018). EUS-guided antegrade metal stenting with hepaticoenterostomy using a dedicated plastic stent with a review of the literature (with video). Endosc. Ultrasound.

[157] Takahara N., Nakai Y., Noguchi K., Suzuki T., Sato T., Hakuta R., Ishigaki K., Saito T., Hamada T., Fujishiro M. (2025). Endoscopic ultrasound-guided hepaticogastrostomy and endoscopic retrograde cholangiopancreatography-guided biliary drainage for distal malignant biliary obstruction due to pancreatic cancer with asymptomatic duodenal invasion: A retrospective, single-center study in Japan. Clin. Endosc..

[158] Nishioka N., Yamamoto Y., Ogura T., Yamada T., Yamada M., Ueno S., Higuchi K. (2020). Risk factors for adverse events associated with bile leak during EUS-guided hepaticogastrostomy. Endosc. Ultrasound.

[159] Oh D., Park D.H., Song T.J., Lee S.S., Seo D.-W., Lee S.K., Kim M.-H. (2016). Optimal biliary access point and learning curve for endoscopic ultrasound-guided hepaticogastrostomy with transmural stenting. Ther. Adv. Gastroenterol..

[160] Nakamura J., Ogura T., Ueno S., Okuda A., Nishioka N., Uba Y., Tomita M., Bessho K., Hattori N., Nishikawa H. (2023). Liver impaction technique improves technical success rate of guidewire insertion during EUS-guided hepaticogastrostomy (with video). Ther. Adv. Gastroenterol..

[161] Ogura T., Higuchi K. (2016). Technical tips for endoscopic ultrasound-guided hepaticogastrostomy. World J. Gastroenterol..

[162] Singh V.K., Dhir V. (2025). Technical Review on Endoscopic Ultrasound-Guided Hepaticogastrostomy. J. Dig. Endosc..

[163] Isayama H., Nakai Y., Itoi T., Yasuda I., Kawakami H., Ryozawa S., Kitano M., Irisawa A., Katanuma A., Hara K. (2019). Clinical practice guidelines for safe performance of endoscopic ultrasound/ultrasonography-guided biliary drainage: 2018. J. Hepato-Biliary-Pancreat. Sci..

[164] Ogura T., Higuchi K. (2021). Technical Review of Developments in Endoscopic Ultrasound-Guided Hepaticogastrostomy. Clin. Endosc..

[165] Alsakarneh S., Madi M.Y., Dahiya D.S., Jaber F., Kilani Y., Ahmed M., Beran A., Abdallah M., Al Ta’ani O., Mittal A. (2024). Is Endoscopic Ultrasound-Guided Hepaticogastrostomy Safe and Effective after Failed Endoscopic Retrograde Cholangiopancreatography?—A Systematic Review and Meta-Analysis. J. Clin. Med..

[166] Hedjoudje A., Pokossy Epée J., Perez-Cuadrado-Robles E., Alric H., Rivallin P., Vuitton L., Koch S., Prat F. (2024). Long-term outcomes of endoscopic ultrasound-guided hepaticogastrostomy in patients with malignant biliary obstruction. United Eur. Gastroenterol. J..

[167] Li J., Tang J., Liu F., Fang J. (2022). Comparison of Choledochoduodenostomy and Hepaticogastrostomy for EUS-Guided Biliary Drainage: A Meta-Analysis. Front. Surg..

[168] Moond V., Loganathan P., Koyani B., Khan S.R., Kassab L.L., Chandan S., Mohan B.P., Broder A., Adler D.G. (2024). Efficacy and safety of EUS-guided hepatogastrostomy: A systematic review and meta-analysis. Endosc. Ultrasound.

[169] Binda C., Dajti E., Giuffrida P., Trebbi M., Coluccio C., Cucchetti A., Fugazza A., Perini B., Gibiino G., Anderloni A. (2024). Efficacy and safety of endoscopic ultrasound-guided hepaticogastrostomy: A meta-regression analysis. Endoscopy.

[170] Tomita M., Ogura T., Hakoda A., Ueno S., Okuda A., Nishioka N., Matsuno J., Hattori N., Nakamura J., Kanadani T. (2026). Prospective evaluation study of EUS-guided hepaticogastrostomy without tract dilation: Comparison to with tract dilation. Endosc. Ultrasound.

[171] Nakai Y., Sato T., Hakuta R., Ishigaki K., Saito K., Saito T., Takahara N., Hamada T., Mizuno S., Kogure H. (2020). Long-term outcomes of a long, partially covered metal stent for EUS-guided hepaticogastrostomy in patients with malignant biliary obstruction (with video). Gastrointest. Endosc..

[172] Facciorusso A., Mangiavillano B., Paduano D., Binda C., Crinò S.F., Gkolfakis P., Ramai D., Fugazza A., Tarantino I., Lisotti A. (2022). Methods for Drainage of Distal Malignant Biliary Obstruction after ERCP Failure: A Systematic Review and Network Meta-Analysis. Cancers.

[173] Amato A., Sinagra E., Celsa C., Enea M., Buda A., Vieceli F., Scaramella L., Belletrutti P., Fugazza A., Cammà C. (2020). Efficacy of lumen-apposing metal stents or self-expandable metal stents for endoscopic ultrasound-guided choledochoduodenostomy: A systematic review and meta-analysis. Endoscopy.

[174] Jang D.K., Lee D.W., Kim S.-H., Cho K.B., Lakhtakia S. (2024). Advances in self-expandable metal stents for endoscopic ultrasound-guided interventions. Clin. Endosc..

[175] Singh S., Kumar V.C.S., Aswath G., Khan H.M.A., Sapkota B., Vinayek R., Dutta S., Dahiya D.S., Inamdar S., Mohan B.P. (2024). Indirect comparison of various lumen-apposing metal stents for EUS-guided biliary and gallbladder drainage: A systematic review and meta-analysis. Gastrointest. Endosc..

[176] Rimbaş M., Anderloni A., Napoléon B., Seicean A., Forti E., Crinò S.F., Tarantino I., Arcidiacono P.G., Fabbri C., Rizzatti G. (2021). Common bile duct size in malignant distal obstruction and lumen-apposing metal stents: A multicenter prospective study. Endosc. Int. Open.

[177] Okuno N., Hara K., Haba S., Kuwahara T., Koda H., Matsumoto S., Ooshiro K., Ogata T. (2025). Clinical Outcomes and Predictors of Early Adverse Events in Primary EUS-Guided Choledochoduodenostomy for Malignant Distal Biliary Obstruction. J. Dig. Endosc..

[178] Li J., Tang J., Fang J., Li Z., Liu F. (2024). Adverse events in endoscopic ultrasound-guided choledochoduodenostomy with lumen-apposing metal stents: A systematic review and meta-analysis. J. Gastroenterol. Hepatol..

[179] Krishnamoorthi R., Dasari C.S., Chandrasekar V.T., Priyan H., Jayaraj M., Law J., Larsen M., Kozarek R., Ross A., Irani S. (2020). Effectiveness and safety of EUS-guided choledochoduodenostomy using lumen-apposing metal stents (LAMS): A systematic review and meta-analysis. Surg. Endosc..

[180] Beunon C., Debourdeau A., Schaefer M., Wallenhorst T., Perez-Cuadrado-Robles E., Belle A., Gonzalez J.-M., Duboc M.C., Caillol F., Toudic H.-P. (2025). Technical failure of endoscopic ultrasound-guided choledochoduodenostomy: Multicenter study on rescue techniques, consequences, and risk factors. Endoscopy.

[181] Khoury T., Sbeit W., Lisotti A., Napoléon B., Fumex F., Marasco G., Eusebi L.H., Fusaroli P., Chan S.M., Shahin A. (2024). Endoscopic ultrasound- versus ERCP-guided primary drainage of inoperable malignant distal biliary obstruction: Systematic review and meta-analysis of randomized controlled trials. Endoscopy.

[182] Fugazza A., Khalaf K., Spadaccini M., Facciorusso A., Colombo M., Andreozzi M., Carrara S., Binda C., Fabbri C., Anderloni A. (2024). Outcomes predictors in endoscopic ultrasound-guided choledochoduodenostomy with lumen-apposing metal stent: Systematic review and meta-analysis. Endosc. Int. Open.

[183] Jacques J., Privat J., Pinard F., Fumex F., Valats J.-C., Chaoui A., Cholet F., Godard B., Grandval P., Legros R. (2018). Endoscopic ultrasound-guided choledochoduodenostomy with electrocautery-enhanced lumen-apposing stents: A retrospective analysis. Endoscopy.

[184] Ogura T., Itoi T. (2021). Technical tips and recent development of endoscopic ultrasound-guided choledochoduodenostomy. DEN Open.

[185] Geyl S., Redelsperger B., Yzet C., Napoleon B., Legros R., Dahan M., Lepetit H., Ginestet C., Jacques J., Albouys J. (2023). Risk factors for stent dysfunction during long-term follow-up after EUS-guided biliary drainage using lumen-apposing metal stents: A prospective study. Endosc. Ultrasound.

[186] Tarantino I., Peralta M., Ligresti D., Amata M., Barresi L., Cipolletta F., Antonio G., Traina M. (2021). Endoscopic ultrasound-guided biliary drainage of malignant stenosis, not treatable with endoscopic retrograde cholangiopancreatography: A single-center, prospective observational study. Endosc. Int. Open.

[187] Vanella G., Leone R., Frigo F., Bronswijk M., van Wanrooij R.L.J., Tamburrino D., Orsi G., Belfiori G., Macchini M., Reni M. (2024). Endoscopic ultrasound-guided choledochoduodenostomy versus hepaticogastrostomy combined with gastroenterostomy in malignant double obstruction (CABRIOLET_Pro): A prospective comparative study. DEN Open.

[188] Giri S., Vaidya A., Kale A., Jearth V., Sundaram S. (2023). Efficacy of lumen-apposing metal stents for the management of benign gastrointestinal stricture: A systematic review and meta-analysis. Ann. Gastroenterol..

[189] Sundaram S., Giri S., Binmoeller K. (2024). Lumen-apposing metal stents: A primer on indications and technical tips. Indian J. Gastroenterol..

[190] Giri S., Harindranath S., Mohan B.P., Jearth V., Varghese J., Kozyk M., Kale A., Sundaram S. (2024). Adverse events with endoscopic ultrasound-guided gastroenterostomy for gastric outlet obstruction—A systematic review and meta-analysis. United Eur. Gastroenterol. J..

[191] Iqbal U., Khara H., Hu Y., Kumar V., Tufail K., Confer B., Diehl D. (2020). EUS-guided gastroenterostomy for the management of gastric outlet obstruction: A systematic review and meta-analysis. Endosc. Ultrasound.

[192] Boghossian M.B., Funari M.P., De Moura D.T.H., McCarty T.R., Sagae V.M.T., Chen Y.-I., Mendieta P.J.O., Neto F.L.P., Bernardo W.M., dos Santos M.E.L. (2021). EUS-guided gastroenterostomy versus duodenal stent placement and surgical gastrojejunostomy for the palliation of malignant gastric outlet obstruction: A systematic review and meta-analysis. Langenbeck’s Arch. Surg..

[193] Tonozuka R., Tsuchiya T., Mukai S., Nagakawa Y., Itoi T. (2020). Endoscopic Ultrasonography-Guided Gastroenterostomy Techniques for Treatment of Malignant Gastric Outlet Obstruction. Clin. Endosc..

[194] Rimbaș M., Lau K.W., Tripodi G., Rizzatti G., Larghi A. (2023). The Role of Luminal Apposing Metal Stents on the Treatment of Malignant and Benign Gastric Outlet Obstruction. Diagnostics.

[195] Abel W.F., Soliman Y.Y., Wasserman R.D., Reddy S., Sangay A.R.V., Monkemuller K.E., Kesar V., Yeaton P., Kesar V. (2024). Endoscopic ultrasound-guided gastrojejunostomy for benign gastric outlet obstruction (GOO): A retrospective analysis of patients and outcomes. Surg. Endosc..

[196] Giri S., Sahu S.K., Khatana G., Gore P., Nath P., Mallick B., Narayan J., Kale A., Sundaram S. (2025). Role of Endoscopic Ultrasound-guided Gastroenterostomy for Benign Gastric Outlet Obstruction. DEN Open.

[197] Govindarajan K.K. (2025). Revisiting malignant gastric outlet obstruction: Where do we stand?. World J. Gastrointest. Endosc..

[198] Bang J.Y., Puri R., Lakhtakia S., Thakkar S., Waxman I., Siddiqui I., Arnold K., Chaudhary A., Mehta S., Singh A. (2025). Endoscopic or surgical gastroenterostomy for malignant gastric outlet obstruction: A randomised trial. Gut.

[199] Ziogas D., Vasilakis T., Kapizioni C., Koukoulioti E., Tziatzios G., Gkolfakis P., Facciorusso A., Papanikolaou I.S. (2024). Revealing Insights: A Comprehensive Overview of Gastric Outlet Obstruction Management, with Special Emphasis on EUS-Guided Gastroenterostomy. Med. Sci..

[200] Ramai D., Nelson R., Chaiyakunapruk N., Ofosu A., Fang J.C. (2025). Endoscopic ultrasound gastroenterostomy vs duodenal stenting for malignant gastric outlet obstruction: Cost-effectiveness study. Endosc. Int. Open.

[201] Arora L., Reddy V.V., Gavini S.K., Chandrakasan C. (2023). Impact of route of reconstruction of gastrojejunostomy on delayed gastric emptying after pancreaticoduodenectomy: A prospective randomized study. Ann. Hepato-Biliary-Pancreat. Surg..

